# Meis2 as a critical player in MN1-induced leukemia

**DOI:** 10.1038/bcj.2017.86

**Published:** 2017-09-29

**Authors:** C K Lai, G L Norddahl, T Maetzig, P Rosten, T Lohr, L Sanchez Milde, N von Krosigk, T R Docking, M Heuser, A Karsan, R K Humphries

**Affiliations:** 1 Terry Fox Laboratory, BC Cancer Agency Research Centre, Vancouver, British Columbia, Canada; 2 Institute of Experimental Hematology, Hannover Medical School, Hannover, Germany; 3 Genome Sciences Centre, BC Cancer Agency Research Centre, Vancouver, British Columbia, Canada; 4 Department of Hematology, Hemostasis, Oncology, and Stem Cell Transplantation, Hannover Medical School, Hannover, Germany; 5 Department of Pathology and Laboratory Medicine, University of British Columbia, Vancouver, British Columbia, Canada; 6 Department of Medicine, University of British Columbia, Vancouver, British Columbia, Canada

## Abstract

Meningioma 1 (MN1) is an independent prognostic marker for normal karyotype acute myeloid leukemia (AML), with high expression linked to all-trans retinoic acid resistance and poor survival. *MN1* is also a potent and sufficient oncogene in murine leukemia models, strongly dependent on the MEIS1/AbdB-like HOX protein complex to transform common myeloid progenitors, block myeloid differentiation, and promote leukemic stem cell self-renewal. To identify key genes and pathways underlying leukemic activity, we functionally assessed MN1 cell phenotypic heterogeneity, revealing leukemic and non-leukemic subsets. Using gene expression profiling of these subsets combined with previously published comparisons of full-length MN1 and mutants with varying leukemogenic activity, we identified candidate genes critical to leukemia. Functional analysis identified *Hlf* and *Hoxa9* as critical to MN1 *in vitro* proliferation, self-renewal and impaired myeloid differentiation. Although critical to transformation, *Meis1* knockdown had little impact on these properties *in vitro*. However, we identified *Meis2* as critical to MN1-induced leukemia, with essential roles in proliferation, self-renewal, impairment of differentiation and disease progression *in vitro* and *in vivo*. Here, we provide evidence of phenotypic and functional hierarchy in MN1-induced leukemic cells, characterise contributions of *Hlf*, *Hoxa9* and *Meis1* to *in vitro* leukemic properties, and reveal *Meis2* as a novel player in MN1-induced leukemogenesis.

## Introduction

Critical to elucidating mechanisms of leukemogenesis is the identification of genes and pathways crucial to leukemic activity. Many such genes have been revealed by their aberrant expression in patient samples or murine leukemia models. Prominent among such genes are numerous members of the HOX transcription factor family, HOX co-factors of the TALE class of Homeobox genes such as *PBX1*, *PBX2* and *MEIS1*, and key upstream regulators of HOX and HOX co-factors such as *MLL*.

Overexpression of the transcriptional co-factor meningioma 1 (*MN1*) has also been observed in a broad spectrum of acute myeloid leukemia (AML)^1, 2, 3, 4, 5^ and has been identified as an independent prognostic marker for AML with normal karyotype. High expression of *MN1* is associated with poor prognosis, shorter overall and relapse-free survival, and poor response to treatment.^3^ In experimental systems, human *MN1* overexpression induces aggressive, fully penetrant AML through the promotion of leukemic cell self-renewal in both human^6^ and murine cells,^7, 8, 9^ impairment of myeloid differentiation,^7, 8^ resistance to all trans retinoic acid-induced differentiation,^8^ and repression of the differentiation-promoting transcription factors C/EBPα and PU.1.^6^ We have previously reported that MN1-induced leukemias are also associated with upregulation of *Hoxa* genes and *Meis1*, as notably apparent when comparing gene expression between MN1 leukemic cells and normal common myeloid progenitors (CMPs), granulocytic-macrophage progenitors, and mature myeloid cells.^10^ Moreover, functional assays have revealed the critical dependence of the MEIS1/AbdB-like HOX protein complex for MN1-induced transformation.^10^ Here, we report findings from further analyses of a range of MN1 leukemia models utilising wild-type and variant forms of MN1 with differing leukemogenic potencies, as well as analysis of gene expression differences associated with phenotypic and functional heterogeneity in MN1 leukemic cells. We identify a wider set of genes relevant to MN1 leukemic activity. Most notable among these is *Meis2*, which we find to be strikingly upregulated in MN1 leukemic cells and essential for MN1 leukemogenic activity in the murine leukemia model. Preliminary analysis of gene expression data from patient samples reinforces the likely and previously unrecognised importance of this additional MEIS family member in human AML. These novel findings complement work identifying *MEIS2* as differentially expressed and functionally critical in RUNX1-RUNX1T1-mediated AML,^11^ possibly extending the relevance of *Meis2* to a range of leukemic subgroups.

## Methods

Detailed methods can be found in Supplementary Materials.

### shRNA viral vectors

shRNA sequences were selected based on previously published sequences^12^ and ordered as non-polyacrylamide gel electrophoresis purified ultramers (Integrated DNA Technologies, Coralville, IA, USA) for PCR amplification and insertion via Gibson assembly into a lentiviral vector with a spleen focus forming virus promoter and miR-E framework for co-expression of the shRNA with a modified monomeric Kusabira Orange 2 fluorescent protein (meKO2).^13^ Primer amplification sequences are provided in Supplementary Table S1 and the shRNA vector (pRRL.PPT.SFFV.meKO2.miR-E.pre*) schematic is provided in Supplementary Figure S3A.

### *In vitro* proliferation assays

Cytokine-dependent cell lines were generated from transduced sorted bone marrow cells or from the cKit^+^ fraction of primary MN1-induced leukemic bone marrow after sorting and cultured in Dulbecco’s Modified Eagle Medium supplemented with 15% fetal bovine serum, 10 ng ml^−1^ human IL6 (hIL6), 6 ng ml^−1^ murine IL3 (mIL3) and 100 ng ml^−1^ murine stem cell factor. For *in vitro* growth and proliferation assays, cells were sorted in triplicate 3 days after shRNA transduction using the BD FACSAria or BD FACSAria Fusion (both from BD Biosciences, San Diego, CA, USA) and counted using the Vi-Cell XR Cell Viability Analyzer (Beckman Coulter, Fullerton, CA, USA). For *in vitro* competitive assays, equal numbers of shRNA-transduced cells and untransduced MN1 cells were sorted by fluorescence-activated cell sorting, and the proportion of meKO2^+^ cells was analysed using the fluorescence-activated cell sorting LSRFortressa (BD Biosciences, San Jose, CA, USA).

### Cell cycle and apoptosis assays

Cells were sorted into triplicate wells by flow cytometry 3 days after shRNA transduction or into phosphate buffered saline (PBS) supplemented with 2% fetal bovine serum (FBS) for immediate analysis. Cell cycle analysis was performed on day 0, 3 and 7 after sorting using the APC BrdU Flow Kit (eBioscience, San Diego, CA, USA) and apoptosis assays were performed 0 and 4 days after sorting using 1 × 10^6^ unsorted cells and the APC Annexin V Apoptosis Detection Kit (eBioscience). Assays were analysed using the FACS LSRFortessa (BD Biosciences, San Jose, CA, USA).

### Bone marrow transplantation and monitoring of mice

Subfractionated or shRNA-transduced bone marrow cells, accompanied by a life-sparing dose of 1 × 10^5^ freshly isolated bone marrow cells from congenic mice, were intravenously injected into irradiated recipient mice (single dose of 810 cGy total-body x-ray irradiation). Engraftment of transduced cells in peripheral blood was monitored every 2–4 weeks as previously described.^14^ Sick or moribund mice were killed and tissues processed as previously described.^14^ C57BL/6J mice were bred and maintained in the Animal Research Centre of the British Columbia Cancer Agency as approved by the University of British Columbia Animal Care Committee (Institutional Animal Care and Use Committee, IACUC) under experimental protocol number A13-0063, and all efforts were made to minimise suffering.

### RNA extraction, cDNA generation, Agilent gene expression array and gene set enrichment analysis

Total RNA was extracted using TRIZOL reagent (Life Technologies, Burlington, Canada) from MN1 cell subpopulations upon euthanasia, from sorted shRNA-transduced MN1 bone marrow cell lines 72 h after transduction, or frozen cell pellets. RNA cleanup was performed using the GeneJET RNA Cleanup and Concentration Micro Kit (ThermoFisher Scientific, Waltham, MA, USA) for gene expression profiling, which was conducted using the Agilent Mouse GE 8x60K microarray (Agilent Technologies, Mississauga, Canada) and analysed as previously described.^15^ cDNA for quantitative real-time PCR was generated and pre-amplified 14 cycles with Taqman PreAmp Mastermix (Applied Biosystems, Foster City, CA, USA) with the relevant primer set diluted 1:100 for global amplification of genes of interest as previously described.^16^

The Broad Institute GSEA software package was used for gene set enrichment analysis.^17^ Gene Ontology sets were obtained from MSigDB v3.1^(ref. 17)^ or gene expression sets from published literature as indicated in the text. Venn diagrams were generated using the BioVenn web application.^18^

### Analysis of human patient samples

The in-house gene expression data set was generated from RNA-Seq data from patients with AML, myelodysplastic syndrome (MDS), therapy-related AML (tAML), therapy-related MDS (tMDS), and AML arising from MDS (AML-MDS). Patients were consented and studies were approved by the BC Cancer Agency Research Ethics Board under protocol number H13-02687. Expression quantification was performed using sailfish (version 0.9.0)^19^ to generate raw read counts and transcripts-per-million expression measures. Variant-calling was performed on gene targets with known relevance to myeloid malignancies using VarScan 2 (version 2.3.9)^20^ and all samples were annotated for insertions-deletions (indels) in NPM1. Expression values of *HOXA9*, *MN1*, *MEIS1* and *MEIS2* were subset from the larger expression matrix and for *MEIS1* and *MN1* divided into high and low expression groups based on median gene expression. The pheatmap program (version 1.0.8) from R (version 3.3.0) was used to cluster all samples by Euclidean distance.

### Statistical analysis

Gene expression analyses were performed by unpaired *t*-tests and applying a Benjamini–Hochberg test correction at a false discovery rate (FDR) of 0.05 using GeneSpring 12.0 (Agilent Technologies).^21^ Functional assays were evaluated using the unpaired Student’s two-tailed *t*-test. Comparisons of survival curves were performed using the Kaplan–Meier method and log-rank test, and analysis of patient RNA-Seq data was performed using a Welch two-sample *t*-test in R (version 3.3.1).^22^ MiSTIC was used to perform single gene and pairwise correlation analyses and visualise Leucegene RNA-Seq data.^23^ Other statistical analyses and visualisation of data were performed with Excel (Microsoft Canada, Mississauga, Canada), GraphPad Prism 6 (GraphPad Software, La Jolla, CA, USA), the Bloodspot database,^24^ and FLOWJO (Tree Star Inc., Ashland, OR, USA). *P*-values less than 0.05 were considered statistically significant.

## Results

### Establishing an experimental framework to explore genes and pathways critical to MN1-induced leukemia

We and others have previously exploited MN1 models of varying leukemic potencies to better understand molecular mechanisms underlying leukemogenesis.^6, 7, 8, 9, 10, 25^ Additionally, our group and others have described phenotypic and functional heterogeneity in MN1 leukemic cells,^6, 7, 8, 9^ suggesting the existence of functionally distinct subpopulations within MN1-induced leukemia. The ability to distinguish between functionally distinct subsets, as well as comparisons of functional and gene expression differences in leukemic cells, have helped identify differentially expressed genes and gene signatures associated with leukemic stem cell (LSC) activity in other AML models.^26, 27, 28, 29, 30^ Thus, to help identify additional genes relevant to MN1-induced leukemia, we performed gene expression comparisons using models with varying leukemic activity: the non-leukemic MN1 variant lacking the N-terminus (MN1Δ1);^14^ MN1 fused to the transcriptional activation domain VP16 (MN1VP16), which induces AML with a longer latency and a more mature myeloid phenotype;^25^ and functionally and phenotypically distinct subpopulations within MN1 leukemic cells, first described in this manuscript. These gene expression data provided an opportunity to search for overlapping genes that may be key to MN1 leukemogenic activity.

### Phenotypic heterogeneity of primary murine MN1 leukemic cells reflects functional heterogeneity

**Figure 1:**
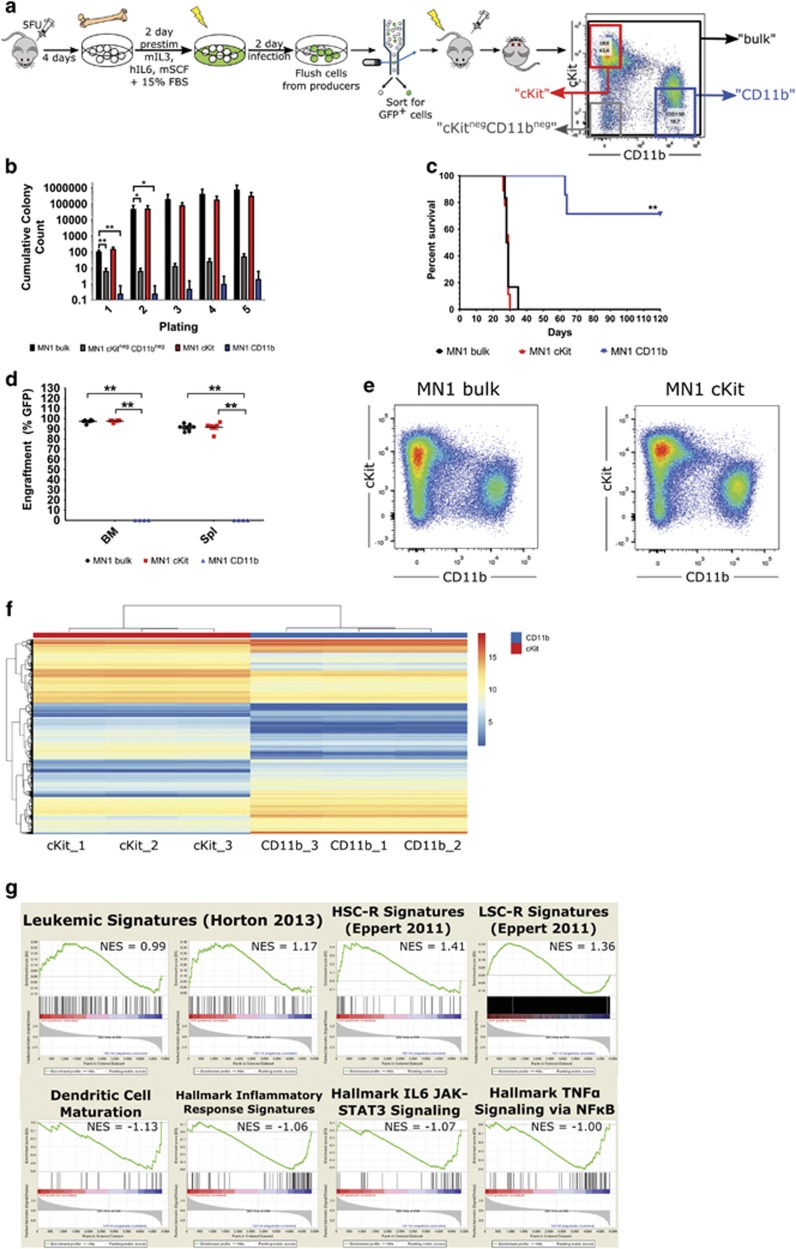
Primary murine MN1 leukemic cells can be separated into phenotypically distinct populations that are functionally heterogeneous. (**a**) Experimental design for generation of MN1-transduced 5-FU bone marrow and fractionation of primary bone marrow from moribund mice into three distinct subpopulations based on the cell surface markers cKit and CD11b (cKit^+^CD11b^−^, ‘cKit’ cKit^neg-mid^CD11b^+^, ‘CD11b’ and cKit^-^CD11b^−^, ‘cKit^neg^CD11b^neg^’) by flow cytometry. (**b**) Serial replating of sorted MN1 bulk, cKit and CD11b bone marrow cells from moribund MN1 mice, represented as cumulative colony counts. Subpopulations from two independent mice, *n*=4; error bars represent ±s.e.m.; *P<0.05, **P<0.01 (unpaired *t*-test versus MN1 bulk). (**c**) Survival curve of mice transplanted with sorted MN1 bulk, cKit, and CD11b bone marrow subpopulations from leukemic mice transplanted with MN1-transduced cells. *n*=6 for MN1 bulk, *n*=8 for cKit and CD11b cells; **P<0.01 (Mantel–Cox). (**d**) Engraftment in bone marrow of moribund/killed secondary mice transplanted with MN1 bulk, cKit and CD11b cells. *n*=6 for MN1 bulk, *n*=8 for cKit and *n*=4 for CD11b. Unpaired two-tailed *t*-test in MN1 bulk versus cKit/CD11b. Error bars represent ±s.e.m.; **P<=0.05. (**e**) Representative flow cytometric analysis of cKit and CD11b cell surface markers on GFP^+^ bone marrow from moribund mice transplanted with MN1 bulk or cKit cells. (**f**) Heatmap of unsupervised hierarchical clustering of the top 500 differentially expressed annotated gene between cKit and CD11b cells. From three mice representing two independent transductions, unpaired *t*-test, fold change ⩾1.5, corrected *P*-value<0.05 (Benjamini–Hochberg correction). (**g**) GSEA of differentially expressed annotated gene sets in cKit versus CD11b cells. NES, normalised enrichment score; FDR, false discovery rate and *P*-value calculated as previously referenced.^17^

Bone marrow from moribund leukemic mice transplanted with cells overexpressing MN1 (MN1 mice) is phenotypically heterogeneous, with cells showing variable expression of the immature cell surface markers cKit and Sca1 and myeloid markers Gr-1 and CD11b.^3^ To determine if this phenotypic heterogeneity is associated with differential leukemic activity, we subfractionated bone marrow from individual moribund MN1 mice into the following subpopulations: cKit^+^CD11b^−^ henceforth known as ‘cKit’ cKit^neg-mid^CD11b^+^ henceforth known as ‘CD11b’ and cKit^-^CD11b^−^ henceforth known as ‘cKit^neg^CD11b^neg^’ (Figure 1a). Functional assessment of these subpopulations’ colony-forming ability reveals that the cKit^neg^CD11b^neg^ and CD11b fractions are essentially devoid of colony-forming cell activity, while the cKit fraction has similar colony-forming ability to MN1 bulk cells (unpaired two-tailed *t*-test, n.s.) (Figure 1b). Colonies derived from the cKit fraction have similar replating ability to bulk MN1 cells over five successive replatings (unpaired two-tailed *t*-test, *P*=0.16). In contrast, the cKit^neg^CD11b^neg^ and CD11b fractions generate significantly fewer colonies with replating ability (unpaired two-tailed *t*-test, *P*<0.05). To investigate the leukemogenic activity of these subpopulations, we transplanted equal numbers of MN1 bulk, cKit and CD11b cells into secondary recipients. Cells from the cKit subpopulation retain full LSC activity, with engraftment levels and median leukemia latency identical to bulk MN1 cells (Mantel–Cox test, n.s.) (Figures 1c and d). Immunophenotyping of bone marrow arising from cKit cells also reveals regeneration of the spectrum of cell types seen in bulk bone marrow from MN1 leukemic mice (Figure 1e, Supplementary Figure S1A). Additionally, mice transplanted with MN1 bulk or cKit cells display splenomegaly, elevated white blood cell numbers, and depressed red blood cell and platelet counts compared to CD11b-transplanted mice (unpaired *t*-test, *P*<0.05 and *P*<0.01) (Supplementary Figures S1B and C). In contrast, mice transplanted with CD11b cells largely fail to develop leukemia after 120 days post-transplant, a significant divergence from bulk MN1-transplanted mouse survival (*P*<0.01, Mantel–Cox test). Most CD11b-transplanted mice show no engraftment of GFP^+^ donor cells (Figure 1d), with GFP^-^ bone marrow containing CD19^+^ B cells, CD4^+^/CD8^+^ T cells, CD11b^+^ monocytes, low expression of immature cKit^+^ cells, few blasts, normal spleen weights, and blood cell counts (Supplementary Figure S1). These data provide support for functional heterogeneity among MN1 leukemic cells and reveal a hierarchical structure consistent with a stem cell model, with the cKit fraction containing leukemia-initiating cell activity and the CD11b subset severely depleted or absent of such cells.

### Gene expression analysis of primary murine MN1 leukemic cell subpopulations supports dichotomous functional identities

Having determined that the cKit and CD11b subpopulations contain and are depleted of leukemia-initiating cell activity, respectively, we performed Agilent microarray mRNA gene expression profiling on matched subpopulations from three leukemic mice from two independent experiments. Analysis of this gene expression data reveals 9796 differentially expressed probe sets or 5516 unique annotated genes with a minimum 1.5-fold difference in expression between the cKit and CD11b subpopulations. Unsupervised hierarchical clustering of the top 500 differentially expressed probes provides evidence for differential gene expression between the two subpopulations (Figure 1f). Furthermore, gene set enrichment analysis reveals that CD11b cells are enriched in genes associated with leukocyte maturation and inflammatory and immune signalling, while the cKit fraction is enriched in genes associated with leukemic,^30^ HSC-related (HSC-R) and LSC-related (LSC-R)^28^ gene signatures (Figure 1g).

### Comparison of additional MN1 gene expression data sets to identify genes potentially relevant to MN1 leukemic ability

**Figure 2:**
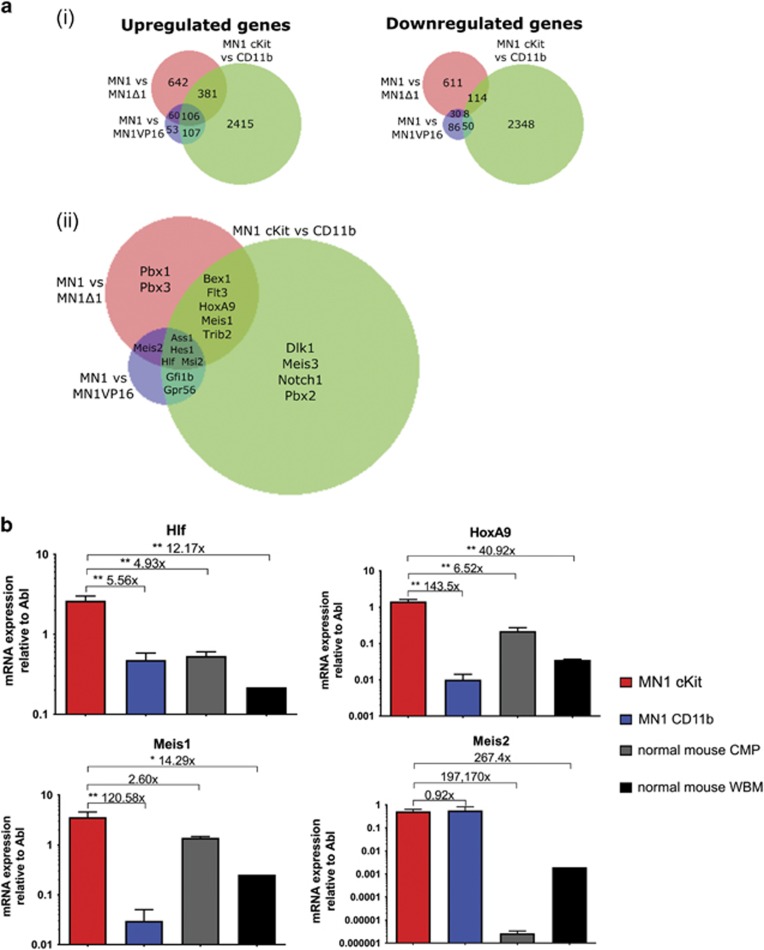
Comparisons of gene expression analysis between MN1 populations with varying leukemic potencies. (**a**) (i) Graphical representation of overlapping differentially expressed up- and downregulated genes between MN1 versus MN1Δ1, MN1 versus MN1VP16 and MN1 cKit versus CD11b data sets. (ii) Comparisons of MN1 gene expression data sets representing models of varying leukemic potencies where shortlisted genes are differentially expressed. (**b**) Absolute gene expression of candidate genes functionally assessed relative to *Abl* in cKit, CD11b, CMP and whole bone marrow (WBM) cells by qRT–PCR. *n*=3 from four mice transplanted with cells from three independent transductions, one-sided ANOVA; error bars represent ±s.e.m.; *P<0.05, **P<0.01.

As a further approach to identify genes important to MN1 leukemic function, we examined gene expression profiles from MN1 murine models with varying leukemic potencies. To the MN1 versus MN1Δ1^(ref. 14)^ and MN1 versus MN1VP16^(ref. 25)^ data sets previously described, we intersected our list of differentially expressed genes between the cKit and CD11b subpopulations. We identified 106 genes upregulated and 8 genes downregulated in all three data sets, while 548 genes were upregulated and 210 genes downregulated in at least two data sets (Figure 2ai). From these analyses, we identified a shortlist of genes for functional validation, guided in our selection by genes previously known to be involved in leukemia, MEIS or HOX co-factor family members, or genes differentially expressed in the same direction in multiple data sets (Figure 2aii).

Expression analysis of the shortlisted genes in cKit and CD11b subpopulations compared to normal murine CMPs, the most differentiated target cell of transformation for MN1 murine leukemias,^10^ and unfractionated mouse bone marrow by qRT–PCR demonstrates that gene expression patterns fall into three categories: genes upregulated in the cKit subpopulation compared to the non-leukemic CD11b subpopulation; genes overexpressed in the CD11b versus cKit subpopulation; and genes similarly expressed in MN1-transduced cells (cKit and CD11b subpopulations) but upregulated compared to normal CMPs and whole bone marrow (Figure 2b and Supplementary Figure S2). From these validated genes, we selected four—*Hlf*, *Hoxa9*, *Meis1* and *Meis2*—that are upregulated in leukemic contexts (cKit over CD11b or MN1 over normal hematopoietic compartments) for functional assessment.

### *Hlf* and *Hoxa9* are critical to MN1 growth and self-renewal properties *in vitro*

**Figure 3:**
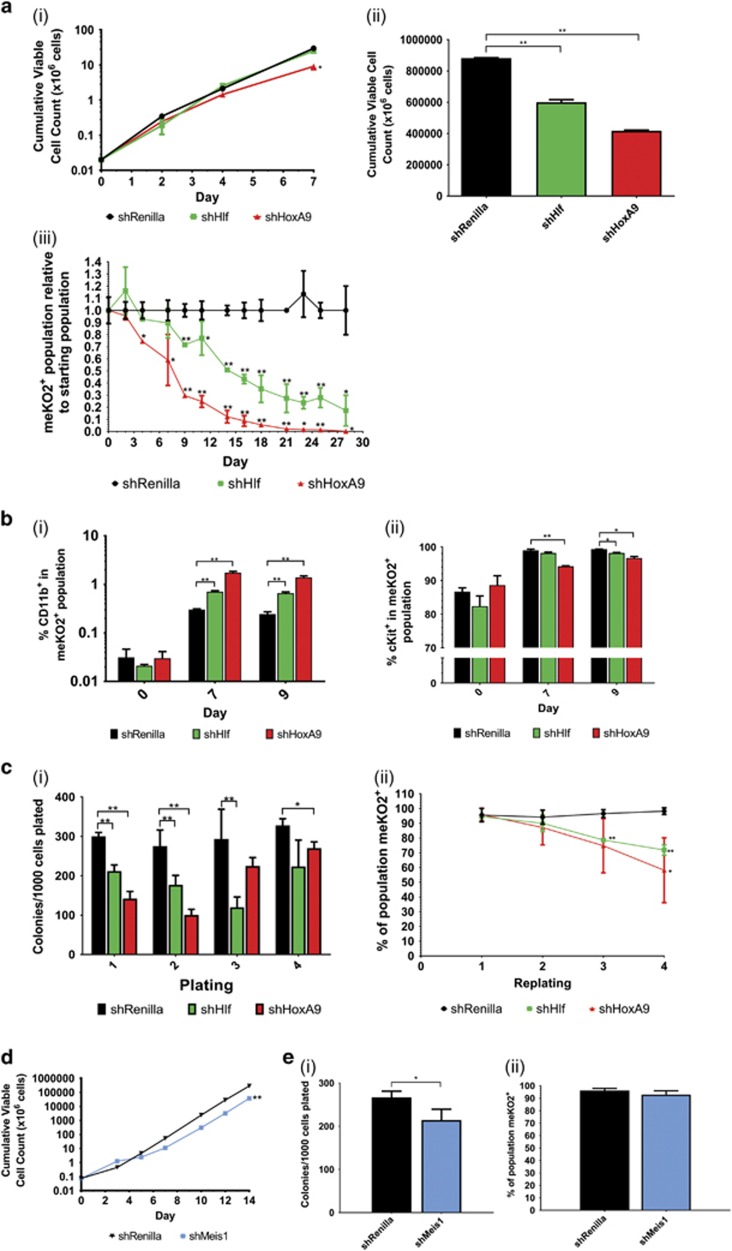
Investigation of the functional relevance of *Hoxa9, Hlf* and *Meis1* on MN1 leukemic properties. (**a**) (i) Growth kinetics of shRenilla-, shHlf- and shHoxa9-transduced MN1 cell lines after flow cytometric sorting. (ii) Cumulative viable cell count of shRenilla-, shHlf- and shHoxa9-transduced MN1 cell lines 14 days after flow cytometric sorting. *n*=3 from three independent experiments, multiple two-tailed *t*-test in shRenilla versus shRNA; error bars represent ±s.d.; *P<0.05, **P<0.01. (iii) Competitive growth assay containing mixed populations of 50% sorted untransduced MN1 cells and 50% sorted shRenilla-, shHlf-, or shHoxa9-transduced (meKO2^+^) MN1 cells, visualised as meKO2^+^ proportion relative to starting population. *n*=3 from three independent experiments, multiple two-tailed *t*-test in shRenilla versus shRNA; error bars represent ±s.d.; *P<0.05, **P<0.01. (**b**) (i) CD11b expression of shRenilla-, shHlf- and shHoxa9-transduced MN1 cell lines 0, 7 and 9 days after flow cytometric sorting. (ii) cKit expression in shRenilla-, shHlf- and shHoxa9-transduced MN1 cells 0, 7 and 9 days after flow cytometric sorting. Sorted meKO2^+^ cells; *n*=3 from four independent experiments, multiple two-tailed *t*-test in shRenilla versus shRNA; error bars represent ±s.d.; *P<0.05, **P<0.01. (**c**) (i) Serial colony replating of shRenilla- and shHlf- and shHoxa9-transduced MN1 cell lines post-sort, represented per 1000 cells plated. (ii) Percentage of meKO2^+^ expressing cells from colony-forming cell assay-derived colonies of shRNA-transduced MN1 cells. Sorted meKO2^+^ cells, *n*=3 from four independent experiments, multiple two-tailed *t*-test; error bars represent ±s.d.; *P<0.05, **P<0.01. (**d**) Growth kinetics of shRenilla-, shMeis1-transduced MN1 cell line after flow cytometric sorting. (**e**) (i) CFU assay of shRenilla- and shMeis1-transduced MN1 cell lines 10 days after flow cytometric sorting. (ii) Percentage of meKO2^+^ expression of cells comprising colonies of transduced MN1 cells in CFU assay. Sorted meKO2^+^ cells; *n*=4 from three independent experiments, represented per 1000 cells plated; multiple two-tailed *t*-test in shRenilla versus shRNA. Error bars represent ±s.e.m.; *P<0.05, **P<0.01.

To functionally assess the roles of the candidate genes in MN1-induced leukemogenesis, we generated optimised lentiviral shRNA vectors expressing a modified monomeric Kusabira Orange (meKO2; Supplementary Figure S3A)^12, 13^ to perform functional assays for *in vitro* proliferation, competitive growth, self-renewal and differentiation in two independently derived MN1 cell lines. Transduced cells stably expressed an established non-targeting control shRNA directed against Renilla luciferase (shRenilla),^12^ or shRNAs against *Hlf* and *Hoxa9* as monitored by meKO2 expression (Supplementary Figure S3Bi). Knockdown efficiencies of 70–72% (shHlf) or 50–63% (shHoxa9) (Supplementary Figure S3Bii) led to significantly impaired MN1 growth kinetics compared to shRenilla-transduced cells within 14 days (unpaired *t*-test, *P*<0.01; Figure 3ai–ii). Major impairments in growth following *Hlf* or *Hoxa9* knockdown is further evident in *in vitro* competition assays, seeded with equal numbers of GFP^+^meKO2^+^ shRNA-transduced MN1 cells and untransduced GFP^+^ MN1 cells, where the contributions of MN1 cells transduced with meKO2^+^ shHlf or shHoxa9 to the total cell population significantly decreases within 7 (unpaired *t*-test, *P*<0.05) and 4 days (unpaired *t*-test, *P*<0.01), respectively, consistent with roles for both genes in MN1 cell growth properties (Figure 3aiii). Additionally, knockdown of *Hlf* or *Hoxa9* results in a slight increase in CD11b^+^ cells after 9 days (unpaired *t*-test, *P*<0.01), accompanied by a brief concordant decrease in cKit expression in shHoxa9 cells, providing evidence for a role of HOXA9 in the characteristic myeloid differentiation block seen in MN1 leukemia (Figure 3b). Knockdown of *Hlf* or *Hoxa9* also results in a significant decrease in colony-forming ability during the first two rounds of serial replating (unpaired *t*-test, *P*<0.01) and decreasing percentages of shRNA-expressing cells comprising these colonies at later timepoints, suggesting selection against shHlf- and shHoxA9-transduced cells (Figure 3c).

### Meis1 is not essential for MN1 leukemic maintenance

Given the significant upregulation of Meis1 in leukemic MN1 cells and subpopulations^14, 25^ and its demonstrated ability to cooperate with MN1 in leukemic transformation of granulocytic-macrophage progenitor,^10^ we assessed the impact of *Meis1* knockdown on MN1 leukemic properties. Unlike *Hlf* and *Hoxa9*, 38% knockdown of *Meis1* (Supplementary Figure S3Ci) results in only mild impairments in cell growth and short-term colony-forming ability (unpaired *t*-test, *P*<0.01) (Figures 3d and e). Of note, there is a marginal decrease in the proportion of cells expressing shMeis1 over the 14 days of the growth assay, as evident by the decrease in meKO2^+^ cells (unpaired *t*-test, *P*<0.05) (Supplementary Figure S3Cii), although there was no effect on *in vitro* competitive ability, CD11b expression or cKit expression (unpaired *t*-test, n.s.) (Supplementary Figures S3Ciii–D). Together, these data suggest that *Meis1* is not required for the maintenance of MN1 cell *in vitro* growth, self-renewal or impairment of differentiation.

### Meis2 is upregulated in immature hematopoietic and MN1 leukemic populations

**Figure 4:**
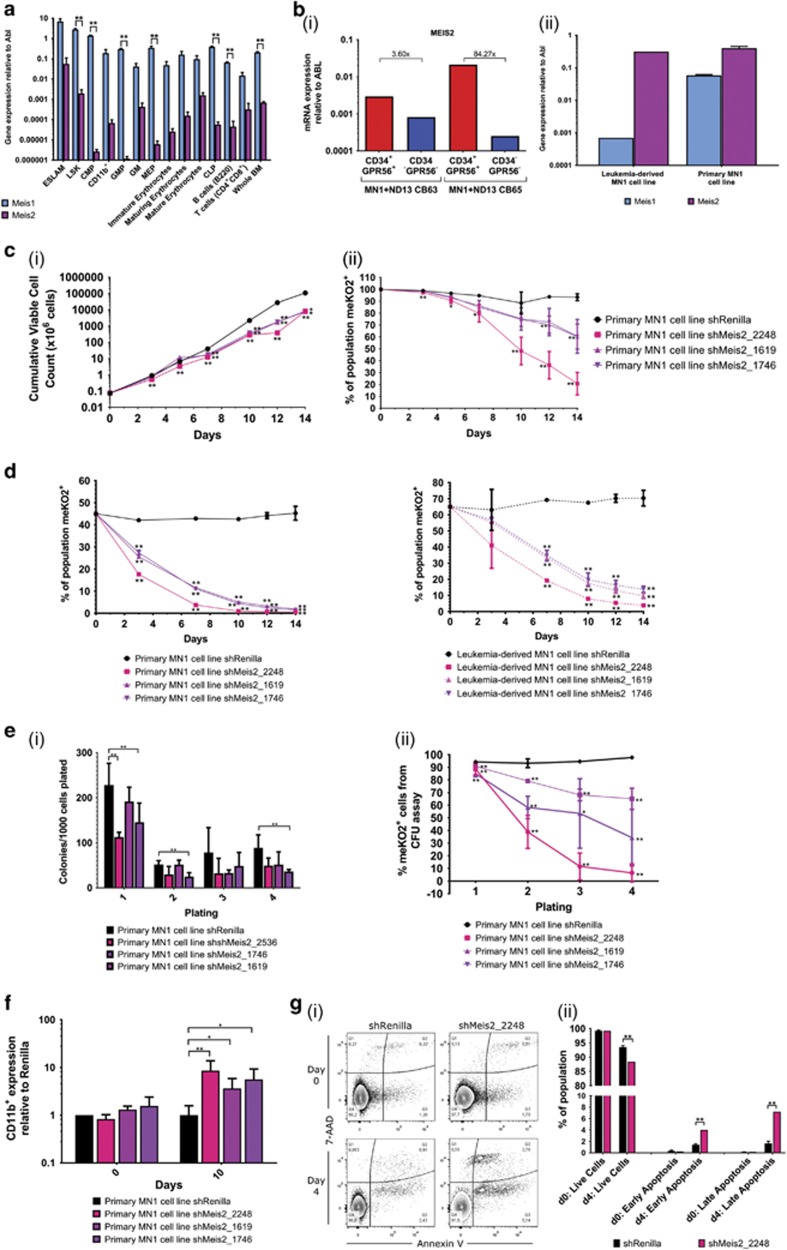
Knockdown of *Meis2* impairs the functional leukemic properties of MN1 cells. (**a**) Gene expression of *Meis1* and *Meis2* relative to *Abl* by qRT–PCR in murine hematopoietic compartments. *n*=3 from three independent mice, two-tailed *t*-test; error bars represent ±s.e.m.; **P<0.01. (**b**) (i) Gene expression of *MEIS2* relative to *ABL* by qRT–PCR in CD34^+^GPR56^+^ compared to CD34^−^GPR56^-^ fraction of two human AML cord blood models generated through overexpression of NUP98-HOXD13 fusion and MN1 (ND13+MN1). *n*=2 from two independent cell lines. (ii) Gene expression of *Meis1* and *Meis2* relative to *Abl* by qRT–PCR in leukemia-derived and primary MN1 cell lines. *n*=3 from each cell line, unpaired two-tailed *t*-test; error bars represent ±s.d. (**c**) (i) Growth kinetics of shRenilla-, shMeis2-transduced MN1 cell line after lentiviral transduction. (ii) Kinetics of meKO2^+^ expression of shRenilla- and shMeis2-transduced MN1 cells after flow cytometric sorting. (**d**) Competitive growth assay containing mixed populations of 50% sorted untransduced MN1 cells and 50% sorted shRenilla- or shMeis2-transduced (meKO2^+^) primary and leukemia-derived MN1 cell lines. Sorted meKO2^+^ MN1 cells; *n*=3 from three (shMeis2_2248) or two (shMeis2_1619 and shMeis2_1746) independent experiments, multiple two-tailed *t*-test in shRenilla versus shRNA; error bars represent ±s.e.m.; *P<0.05, **P<0.01. (**e**) (i) Serial colony replating of shRenilla- and shMeis2-transduced sorted MN1 cell line, represented per 1000 cells plated. (ii) Percentage of meKO2^+^ expressing cells comprising colonies of transduced MN1 cells in CFU assay. Sorted meKO2^+^ cells, *n*=4 from two independent experiments, multiple two-tailed *t*-test; error bars represent ±s.e.m.; *P<0.05, **P<0.01. (**f**) (i) CD11b^+^ expression of shRenilla- and shMeis2-transduced leukemia-derived MN1 cell lines 10 days after flow cytometric sorting. Sorted meKO2^+^ MN1 cells; *n*=3 from three (shMeis2_2248) or two (shMeis2_1619 and shMeis2_1746) independent experiments; error bars represent ±s.e.m.; *P<0.05, **P<0.01. (**g**) (i) Representative flow cytometric analysis of apoptosis (Annexin V/7-AAD staining) of shRenillla- and shMeis2-transduced MN1 cells at 0 and 4 days post-sort. (ii) Annexin V apoptosis assay summary of shRenilla- and shMeis2-transduced live, early apoptotic and late apoptotic MN1 cells at 0 and 4 days post-sort from three independent experiments in triplicate. Multiple two-tailed *t*-test in shRenilla versus shMeis2_2248. Error bars represent ±s.e.m.; **P<0.01.

Given the prevalence of MEIS1 overexpression in AML,^31, 32, 33, 34^ its critical role in MN1 leukemic transformation,^10^ and its upregulated gene expression in the two MN1 leukemic versus non-leukemic data sets examined (MN1 versus MN1Δ1 and cKit versus CD11b),^14^ the minimal effects of *Meis1* knockdown on growth, self-renewal, and impairment of differentiation stimulated closer examination of a possible role for the MEIS family member MEIS2. *Meis2* is expressed at significantly lower levels than *Meis1* in normal hematopoietic cell compartments although, like *Meis1*, it is expressed at higher levels in more immature subsets (Figure 4a). In primary MN1 leukemic cells, although *Meis2* is equally expressed between the MN1 cKit and CD11b subpopulations (unpaired *t*-test, n.s.), expression in these subsets is upregulated by several orders of magnitude compared to murine CMPs (unpaired *t*-test, *P*<0.05) and over 200-fold over whole bone marrow (unpaired *t*-test, *P*<0.05) (Figure 2b). Further highlighting *Meis2* is its previous identification as among the top-ranked genes upregulated between both MN1 and MN1Δ1^(ref. 14)^ and MN1 and MN1VP16.^25^ Moreover, *MEIS2* is upregulated in the leukemia-initiating cell-containing CD34^+^GPR56^+^^(ref. x35)^ fraction of a human cord blood model of AML characterised by overexpression of *MN1* and *NUP98-HOXD13*^(ref. 15)^ (Figure 4bi), as well as two independently derived MN1 murine cell lines (Figure 4bii). Together, these data suggest that MN1 leukemia is associated with substantial upregulation of *Meis2* and this upregulation may play a key role in MN1 leukemia.

### Knockdown of Meis2 critically impairs MN1 cell growth, self-renewal and impairment of differentiation *in vitro*

To test the effect of *Meis2* knockdown on MN1 leukemia, we used two different murine models. The leukemia-derived MN1 cell line is a leukemic cell line established from *in vitro* culture of cKit^+^ primary MN1 bone marrow cells. The second model was generated by culturing 5-FU-treated bone marrow cells retrovirally transduced with MN1 to establish a primary leukemic MN1 cell line. Three shRNAs^12^ against *Meis2* provide gene knockdown individually ranging from 26 to 54% as assessed by qRT–PCR analysis 3 and 6 days post-transduction (unpaired *t*-test, *P*<0.01) (Supplementary Figure S3E). In both models, transduction with each of the three *Meis2* shRNAs significantly impairs cell growth, apparent as early as 5 days after plating (unpaired *t*-test, *P*<0.01) and resulting in an average of 16-fold fewer cells after 14 days (Figure 4ci, Supplementary Figure S4Ai). Cells transduced with shMeis2 also show a decreasing proportion of shMeis2 vector over the 14 days of culture, as assessed by meKO2 expression (unpaired *t*-test at day 14, *P*<0.01) (Figure 4cii, Supplementary Figure S4Aii), suggesting cells with downregulated *Meis2* are at a competitive disadvantage and are rapidly eliminated from the population. This is supported by *in vitro* competitive assays, in which the contribution of shMeis2-transduced MN1 cells decreases rapidly, with significantly fewer cells than their untransduced counterparts within 7 days (unpaired *t*-test, *P*<0.01), indicative of severe growth impairment *in vitro* (Figure 4d). To measure the effect of *Meis2* knockdown on *in vitro* self-renewal ability, we functionally assayed transduced cells for colony-forming ability. Cells transduced with shMeis2 show impairments in colony formation over four successive platings in the colony-forming cell assay (unpaired *t*-test, *P*<0.05 or *P*<0.01), providing evidence for impaired *in vitro* self-renewal upon knockdown of *Meis2* (Figure 4ei, Supplementary Figure S4Bi). Importantly, the proportion of cells comprising these colonies that express the shMeis2, measured by proportion of meKO2^+^ cells, drastically decreases compared to shRenilla-transduced control cells in secondary platings and beyond (unpaired *t*-test, *P*<0.01) (Figure 4eii, Supplementary Figure S4Bii). This significant decrease suggests that the numbers of colonies produced by shMeis2-transduced MN1 cells is an overestimate of their colony-forming ability, and is thus consistent with rapid removal of MN1 leukemic cells upon downregulation of *Meis2*. Moreover, *Meis2* knockdown leads to increases in CD11b (unpaired *t*-test, *P*<0.01 for leukemia-derived MN1 cell line, *P*<0.05 for primary MN1 cell line) (Figure 4f, Supplementary Figure S4Ci) and, to a lesser degree, Gr-1 expression *in vitro* (unpaired *t*-test, n.s.) (Supplementary Figure S4Cii), suggesting that Meis2 may also contribute to the myeloid differentiation block. Together, these data suggest that there is a strong negative selection against MN1 cells lacking upregulated *Meis2* expression, with knockdown of *Meis2* impairing *in vitro* growth, self-renewal ability and survival, and the myeloid differentiation block characteristic of MN1 leukemic cells.

### Knockdown of Meis2 increases apoptosis of MN1 cells

To further investigate the impairment seen in MN1 cell growth and proliferation upon knockdown of *Meis2*, we examined the effect of *Meis2* downregulation on cell cycle and apoptosis. Studies in other tissues have pointed to a regulatory role for *MEIS2* at G2-M cell cycle checkpoints^36^ and in S phase.^37^ However, BrdU assays show no changes in MN1 cell cycle distribution upon knockdown of *Meis2* (Supplementary Figure S4D). In contrast, apoptosis assays based on Annexin V binding show significant increases in both early and late apoptosis, with an 8.1±0.3% increase in total apoptotic cells after 4 days in culture (Figure 4g). Concurrently, a 5.1±1.1% decrease in the proportion of live cells is observed, suggesting a negative selection pressure against loss of *Meis2* in MN1 cells that results in their rapid removal from the population (unpaired *t*-test, *P*<0.01). Together, these data provide new evidence that *Meis2* contributes to the block in *in vitro* apoptosis characteristically seen in MN1 leukemia.

### Knockdown of Meis2 impairs MN1 leukemic cell engraftment kinetics *in vivo*

**Figure 5:**
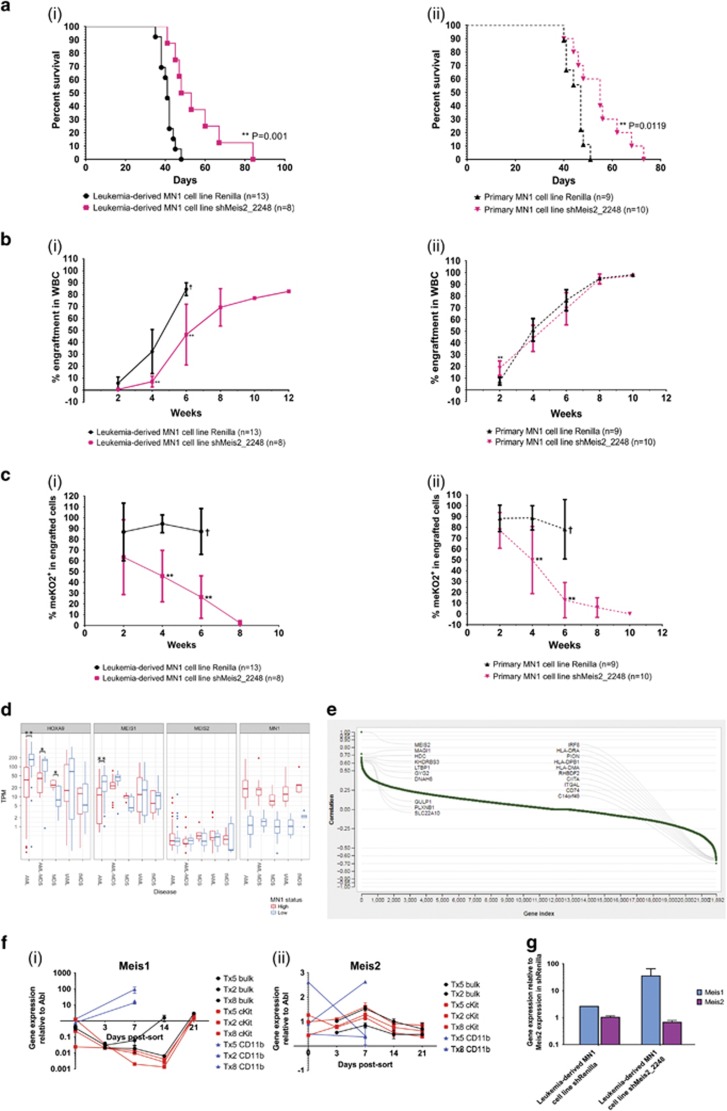
Knockdown of *Meis2* increases latency and delays engraftment kinetics of MN1 cells due to loss of shMeis2-transduced cells. (**a**) Survival curve of mice transplanted with shRenilla- and shMeis2-transduced (i) leukemia-derived and (ii) primary MN1 cell lines. Leukemia-derived: *n*=13 for shRenilla, *n*=8 for shMeis2; primary: *n*=9 for shRenilla, *n*=10 for shMeis2; Mantel–Cox test. (**b**) Engraftment kinetics of mice transplanted with shRenilla- and shMeis2-transduced (i) leukemia-derived and (ii) primary MN1 cell lines, as determined by bi-weekly peripheral blood analysis. Leukemia-derived: *n*=13 for shRenilla, *n*=8 for shMeis2; primary: *n*=9 for shRenilla, *n*=10 for shMeis2; multiple two-tailed *t*-test in shRenilla versus shMeis2; error bars represent ±s.d.; † indicates all mice were killed after this timepoint due to disease, *P<0.05, **P<0.01. (**c**) Proportion of meKO2^+^ cells within engrafted cells of mice transplanted with shRenilla- and shMeis2-transduced (i) leukemia-derived and (ii) primary MN1 cell lines, as determined by bi-weekly peripheral blood analysis. Leukemia-derived: *n*=13 for shRenilla, *n*=8 for shMeis2; primary: *n*=9 for shRenilla, *n*=10 for shMeis2. Multiple two-tailed *t*-test in shRenilla versus shMeis2. Error bars represent ±s.d.; † indicates all mice were killed after this timepoint due to disease, *P<0.05, **P<0.01. (**d**) Distribution of *HOXA9*, *MEIS1*, *MEIS2* and *MN1* expression in patients with AML or MDS, categorised by *MN1* expression level. *P<0.05, **P<0.01 (two-sided Welch’s two-sample *t*-test). (**e**) Waterfall plot visualising single gene correlation of *MEIS2* and top 10 positively and negatively correlated genes based on RNA-Seq from Leucegene normal karyotype AML data set. (**f**) (i) *Meis1* and (ii) *Meis2* gene expression kinetics of primary MN1 mouse bone marrow cells sorted into MN1 bulk, cKit and CD11b subpopulations and cultured *in vitro*. *n*=3 from three independent experiments. Error bars represent ±s.d. (**g**) Relative gene expression of *Meis1* and *Meis2* 6 days post-transduction in shRenilla- or shMeis2-transduced MN1 cells. Sorted meKO2^+^ cells, *n*=3, unpaired *t*-test. Error bars represent ±s.d.; *P<0.05, **P<0.01.

To evaluate the role of *Meis2* in engraftment and leukemogenicity of MN1 cells, we transplanted 1 × 10^5^ shRenilla- or shMeis2-transduced MN1 cells into lethally irradiated recipient mice. Knockdown of *Meis2* significantly increases the latency of disease in both MN1 leukemic models, with median latency of disease onset increasing from 41 to 50 days in the leukemia-derived MN1 model (Mantel–Cox, *P*=0.001) and from 47 to 55 days in the primary MN1 cell line model (Mantel–Cox, *P*=0.0119) (Figure 5a). Analysis of engraftment kinetics in peripheral blood also reveals significant impairments in the ability of shMeis2-transduced cells to engraft at early timepoints (Figure 5b), and is thus consistent with delayed leukemia onset. This delay is especially prominent in the first 6 weeks post-transplant, as mice transplanted with shMeis2-transduced cells display significantly lower levels of engraftment 4 weeks post-transplant (unpaired *t*-test, *P*<0.01). During the first 6 weeks, mice transplanted with shMeis2-transduced cells also show modest but insignificant increases in Gr-1^+^, Gr-1^+^CD11b^+^, and CD11b^+^ cells and decreases in cKit^+^ cells (all n.s.), suggesting that decreased *Meis2* alone is insufficient to relieve the block in myeloid differentiation *in vivo* (Supplementary Figures S5A–D). At time of euthanasia, engraftment levels had plateaued and signs of leukemia (high proportion of donor-derived cells, splenomegaly, elevated white blood cell counts, depressed red blood cell and platelet counts) were similar between experimental and control conditions (Supplementary Figures S5E and F). However, examination of the proportion of meKO2^+^ cells within engrafted donor cells shows markedly fewer shMeis2-transduced cells compared to shRenilla-transduced control cells, demonstrating strong selection against cells with downregulated *Meis2* (Figure 5c). Together, these data demonstrate that *Meis2* is critical to MN1 *in vivo* leukemogenic ability, with knockdown severely compromising engraftment kinetics, increasing time to onset of disease and rapid depletion of shMeis2-transduced cells from the population.

### Exploring the relationship between MEIS1, MEIS, and MN1

Previous studies with patient samples have identified an inverse relationship between HOXA and HOXB cluster genes and MEIS1 compared to MN1.^38^ However, upregulation of *HOXA9* also correlates to high *MN1* expression in AML patients with complex karyotype/loss of chromosome 5 or 7.^39^ As upregulation of *MEIS2* was recently reported in the context of patients with the RUNX1-RUNX1T1 translocation,^11^ we were interested in further assessing relationships between MEIS1, MEIS2 and MN1. Analysis of an in-house data set comprised of patients with AML, MDS, therapy-related AML (tAML) or therapy-related MDS (tMDS), AML subsequent to MDS was consistent with an inverse correlation between *HOXA9* and *MEIS1* expression compared to expression of *MN1* (Welch’s two-sample *t*-test, *P*<0.05) (Figure 5d). Additionally, there is a wide range of *MEIS2* levels in patients with lower *MN1* expression, although, interestingly, some patients with high *MEIS2* expression also exhibit lower *MEIS1* expression. However, the number of patients in this category is insufficient to determine statistical significance (data not shown).

To investigate if *MEIS2* upregulation could be detected in broader data sets of malignant hematopoietic subsets, we examined the TCGA AML and Leukemia MILE data sets.^24^ These larger data sets show upregulation of *MEIS2* in both AML characterised by inv^16^ and RUNX1-RUNX1T1^(ref. 24)^ (Supplementary Figure S6A). As high *MN1* is associated with inv^16^ we hypothesise there is a subset of patients who display inv^16^ with both high *MN1* and *MEIS2* expression. Intriguingly, data from patients with normal karyotype AML (*n*=48) from the Leucegene RNA-Seq data set^23^ show that *IRF8*, which is repressed by MN1,^25^ is among the top 10 genes negatively correlated with *MEIS2* expression (*r*=−0.61) (Figure 5e, Supplementary Figure S6B), Additionally, many of the other top 10 negatively correlated genes have important roles for immune function and thus, underline the immune suppressive function of MN1 signalling.

Gene expression from Valk and colleagues^2^ show no significant correlation between *MEIS2* and AML characterised by inv^16^ or high *MN1* expression. However, across AML karyotypes, *MEIS1* and *MEIS2* are inversely and significantly correlated (Tables 1, 2, 3). As MEIS1 and MEIS2 are highly similar at the amino acid level, these family members may share some downstream targets. Gene expression kinetics of sorted MN1 subpopulations from secondary transplantation murine leukemic bone marrow show that low levels of *Meis1* in the first 7–14 days in culture are accompanied by increases in *Meis2* expression, which is reversed as *Meis1* levels increase after 14 days in culture (Figure 5f). Furthermore, knockdown of *Meis2* in our leukemia-derived MN1 cell line results in an approximately 10-fold increase in *Meis1* expression (unpaired *t*-test, n.s.), supporting compensatory expression between these family members (Figure 5g).

## Discussion

In this study, we sought to identify and characterise additional genes underlying leukemias derived from overexpression of the potent oncogene MN1. We demonstrate that MN1 murine leukemic cells reflect a phenotypic and functional hierarchy in which LSCs reside predominantly in the cKit subset and can regenerate the full spectrum of phenotypic and functional heterogeneity. Gene expression profiling of these MN1 subpopulations, combined with comparisons of cells transduced with wild-type MN1 versus variants with differing leukemic activity, identified a shortlist of genes differentially expressed in multiple data sets that are potentially critical to MN1-induced leukemogenesis. Among these genes, we focused on a subset including genes previously known to be involved in cancer or leukemia, HOX and MEIS family co-factors, and genes differentially expressed in multiple comparisons. Knockdown of *Hlf* or *Hoxa9* significantly blunts leukemic cell growth and colony formation *in vitro*, demonstrating key roles of these genes in these *in vitro* MN1 leukemic properties. Surprisingly, *Meis1* knockdown has minimal effects on *in vitro* measures of leukemic activity. In contrast, *Meis2* is instrumental for the maintenance of MN1 cells. Knockdown of *Meis2* profoundly impairs *in vitro* proliferation and colony-forming ability, owed in part to increased apoptosis, and partially restores myeloid differentiation ability. Transplantation of shMeis2-transduced MN1 cells increases the latency of disease onset due to delayed engraftment kinetics and rapid depletion of shMeis2-expressing cells during leukemic development. Together, we provide further support for the roles of *Hoxa9* and *Meis1* in leukemic maintenance, demonstrate a functional role for *Hlf* in AML, and identify *Meis2* as a novel, essential player in MN1-induced leukemogenesis.

Previous literature has utilised phenotypic heterogeneity in leukemic cells to demonstrate corresponding functional heterogeneity, providing insight into target cells of transformation and mechanisms of leukemic activity.^27, 29, 35^ Furthermore, demonstrable heterogeneity within a leukemic population has been critical to the identification of gene signatures relevant to leukemic development.^26, 28, 30^ For the first time, we demonstrate phenotypic and functional heterogeneity in MN1 leukemic cells and exploit this and other MN1 expression models to identify genes differentially regulated between MN1 models of varying leukemic activity. Among genes differentially expressed in two or more data sets are *Hoxa9* and *Meis1*, known for their role in leukemic transformation, self-renewal, proliferation and differentiation impairment;^40, 41, 42, 43, 44^ *Hlf*, a key player in HSC engraftment, apoptosis inhibition and B-ALL;^45, 46^ *Hes1*, linked to FLT3 and *Notch* signalling in AML, themselves highlighted genes in leukemia;^47^ and *Gpr56*, recently identified to mark primary human AML cells with high repopulating potential.^35^ Interestingly, we also identify genes differentially expressed between MN1 and normal blood subpopulations, most notably *Meis2*, which was found to be critical to MN1 leukemic activity.

Previous work has identified MEIS1 as essential for leukemic transformation^10^ and maintenance.^48^ Co-engineered over-expression of *MEIS1* and *Hoxa9* in granulocytic-macrophage progenitor cells renders them susceptible to MN1 transformation and ChIP-Seq analysis has documented striking overlap between the genomic binding sites of MN1 and MEIS1.^10^ It was thus intriguing that knockdown of *Meis1* had only minor effects on *in vitro* growth kinetics and short-term colony-forming ability of MN1 cells and no effect on *in vitro* competitive ability or myeloid differentiation block. These findings are consistent with MN1 requiring upregulation of *Meis1* for disease initiation but not maintenance, although the possibility remains that the magnitude of *Meis1* downregulation achieved was insufficient to block the effects of *Meis1*.

Closer examination of other MEIS family members also identify *Meis2* as significantly upregulated in MN1 cells compared to MN1Δ1^(ref. 14)^ or MN1VP16-transduced cells,^25^ and upregulated in MN1 leukemic cells over normal CMPs and bone marrow, as well as the leukemia-initiating cell-containing fraction of a human cord blood model of MN1-induced leukemia.^15^ This raises the interesting possibility that *Meis2*, rather than *Meis1*, is most critical to MN1 leukemogenic activity, a possibility further supported by reports of upregulated *Meis2* in AML and ALL cell lines,^49^ a murine AML model driven by co-overexpression of *Hoxa9* and *Meis1*,^50^ and patient samples and human cell lines characterised by the RUNX1-RUNX1T1 translocation.^11^

Supporting this, knockdown of *Meis2* in MN1 leukemic cell lines results in a significant impairment in growth kinetics and *in vitro* self-renewal. This is consistent with the role of high *Meis2* expression in proliferation and regulation of fate specification in retinal progenitor cells,^51^ as well as decreased proliferation and colony-forming ability upon *MEIS2* knockdown in RUNX1-RUNX1T1 containing cell lines.^11^

Knockdown of *Meis2* in leukemic cells is also associated with a shift to more differentiated cells expressing CD11b *in vitro* and increased mature myeloid cells (Gr-1^+^, CD11b^+^, Gr-1^+^CD11b^+^) during the first 6 weeks post-transplantation *in vivo*. Similarly, downregulation of *Meis2*, which is normally expressed with *Meis1*, *Hoxa9* and *Hoxb4* in undifferentiated 32Dcl3 cells, is required for 32Dcl3 cell differentiation in the presence of Il3 and G-CSF.^52^ Additionally, knockdown of *MEIS2* in RUNX1-RUNX1T1 containing cell lines show decreased proliferation and colony-forming ability and increased CD11b expression,^11^ supporting a role for MEIS2 in the maintenance of immature hematopoietic cells.

We also documented that downregulation of *Meis2* leads to increases in the proportion of cells in early and late apoptosis. These observations are consistent with examinations of embryonic lethal *Meis2* knockout mice, which exhibit large-scale cellular destruction and apoptosis,^53^ and the marked decrease in cell viability following siMEIS2 depletion in a primary patient sample characterised by the RUNX1-RUNX1T1 translocation.^11^ This is also consistent with the marked impairment in leukemogenic activity of transplanted MN1 cells *in vivo* combined with the rapid depletion of shMeis2-transduced MN1 cells immediately following transplantation observed, suggesting that MN1 cells expressing shMeis2 are rapidly removed from the *in vivo* environment through a combination of apoptosis, terminal differentiation, and an inability to compete with untransduced MN1 cells.

MEIS1 and MEIS2 share 85.7% identical amino acid sequence, with nearly identical DNA binding and transcriptional activation domains.^54^ Consequently, these protein family members may bind to similar DNA sequences and likely overlap in some target genes. *Meis1* conditional knockout mice generate all hematopoietic compartments, albeit at lower cell numbers,^16, 55, 56^ suggesting that other transcriptional pathways, and notably *Meis2*, can compensate for the loss of *Meis1*. Consistent with this, we noted that expression levels of *Meis2* in MN1 cKit subsets isolated from MN1 leukemic bone marrow increase over the first 7 days in culture, whereas *Meis1* expression levels decrease concurrently. This inverse expression of *Meis1* and *Meis2* suggests a degree of redundancy, such that upregulation of one MEIS family member—typically MEIS1—may be sufficient for leukemogenesis. This is supported by data from the human acute myelomonocytic ML2 cell line, which has substantial expression of *MEIS2* compared to *MEIS1*, in which knockdown of *MEIS1* has no effect on *in vitro* clonogenic ability.^48^ In addition, Valk and colleagues report that *MEIS1* and *MEIS2* are inversely and significantly correlated across AML subtypes,^2^ providing support for a compensatory relationship between *MEIS1* and *MEIS2* where AML may only require upregulation of one MEIS family member, typically *MEIS1*. Interestingly, the TCGA AML data set shows that high *MEIS2* expression is associated with improved overall survival, suggesting that *MEIS2* may serve as a novel clinical prognostic marker, and that upregulation may also activate sufficient overlapping pathways to induce less-aggressive AML. Consistent with this idea, knockdown of *Meis2* in our leukemia-derived MN1 cell line also shows concurrent upregulation of *Meis1*. However, this did not mediate a growth advantage or rescue the phenotype, suggesting that not all functions are shared between the two proteins. This provides evidence that the expression of *MEIS1* and *MEIS2* may be modulated to the activity of other family members and provides impetus to develop models to further investigate differential regulation of MEIS family members in a leukemic context.

In summary, these models provide a platform to identify and functionally assess genes critical to MN1 leukemic activity. We describe a phenotypic and functional hierarchy in MN1 leukemic cells and, through comparisons of gene expression profiling of these subpopulations with existing expression data sets of MN1 models with varying leukemic activity, identify a subset of genes potentially relevant to MN1 leukemic activity. These genes include *Hoxa9*, *Hlf* and *Meis2*, which were functionally validated, although there are likely additional genes to be found within the MN1 gene expression comparisons reported here. The novel discovery of *Meis2* as critical to MN1-induced leukemic activity compels further investigation to unravel the basis for the profound upregulation of *Meis2* in MN1 leukemias, delineate potential functional differences between *MEIS2* and *MEIS1*, and stimulates further study into the role of MEIS2 in additional leukemic settings.

## Figures and Tables

**Table 1:** Correlation of MN1, MEIS1, MEIS2 and MEIS3 gene expression in patients with AML

*Pearson correlations*								
		*MN1*	*MEIS1_p1*	*MEIS1_p2*	*MEIS1_p3*	*MEIS2*	*MEIS3_p1*	*MEIS3_p2*
MN1	Pearson correlation	1	−0.015	−0.006	−0.043	−0.026	0.007	−0.104
	Sig. (two-tailed)		0.798	0.915	0.459	0.653	0.9	0.077
	*N*	293	293	293	293	293	293	293
MEIS1_p1	Pearson correlation	−0.015	1	0.972**	0.924**	−0.214**	0	−0.05
	Sig. (two-tailed)	0.798		0	0	0	0.994	0.396
	*N*	293	293	293	293	293	293	293
MEIS1_p2	Pearson correlation	−0.006	0.972**	1	0.894**	−0.226**	0.016	−0.053
	Sig. (two-tailed)	0.915	0		0	0	0.784	0.37
	*N*	293	293	293	293	293	293	293
MEIS1_p3	Pearson correlation	−0.043	0.924**	0.894**	1	−0.189**	0.01	−0.023
	Sig. (two-tailed)	0.459	0	0		0.001	0.86	0.692
	*N*	293	293	293	293	293	293	293
MEIS2	Pearson correlation	−0.026	−0.214**	−0.226**	−0.189**	1	0.064	0.005
	Sig. (two-tailed)	0.653	0	0	0.001		0.274	0.936
	*N*	293	293	293	293	293	293	293
MEIS3_p1	Pearson correlation	0.007	0	0.016	0.01	0.064	1	−0.101
	Sig. (two-tailed)	0.9	0.994	0.784	0.86	0.274		0.084
	*N*	293	293	293	293	293	293	293
MEIS3_p2	Pearson correlation	−0.104	−0.05	−0.053	−0.023	0.005	−0.101	1
	Sig. (two-tailed)	0.077	0.396	0.37	0.692	0.936	0.084	
	*N*	293	293	293	293	293	293	293

** Correlation is significant at the 0.01 level (two-tailed).

**Table 2:** Correlation of MN1, MEIS1, MEIS2 and MEIS3 gene expression in patients with normal karyotype AML

*Pearson correlations*								
		*MN1*	*MEIS1_p1*	*MEIS1_p2*	*MEIS1_p3*	*MEIS2*	*MEIS3_p1*	*MEIS3_p2*
MN1	Pearson correlation	1	0.091	0.095	0.041	−0.062	0.044	0.061
	Sig. (two-tailed)		0.292	0.271	0.637	0.475	0.61	0.484
	*N*	135	135	135	135	135	135	135
MEIS1_p1	Pearson correlation	0.091	1	0.0967**	0.912**	−0.244**	0.052	−0.145
	Sig. (two-tailed)	0.292		0	0	0.004	0.546	0.094
	*N*	135	135	135	135	135	135	135
MEIS1_p2	Pearson correlation	0.095	0.967**	1	0.874**	−0.237**	0.08	−0.158
	Sig. (two-tailed)	0.271	0		0	0.006	0.356	0.067
	*N*	135	135	135	135	135	135	135
MEIS1_p3	Pearson correlation	0.041	0.912**	0.874**	1	−0.245**	0.065	−0.095
	Sig. (two-tailed)	0.637	0			0.006	0.356	0.067
	*N*	135	135	135	135	135	135	135
MEIS2	Pearson correlation	−0.062	−0.244**	−0.237**	−0.245**	1	−0.052	0.102
	Sig. (two-tailed)	0.475	0.004	0.006	0.004		0.549	0.24
	*N*	135	135	135	135	135	135	135
MEIS3_p1	Pearson correlation	0.044	0.052	0.08	0.065	−0.052	1	−0.081
	Sig. (two-tailed)	0.61	0.546	0.356	0.455	0.549		0.353
	*N*	135	135	135	135	135	135	135
MEIS3_p2	Pearson correlation	0.061	−0.145	−0.158	−0.095	0.102	−0.081	1
	Sig. (two-tailed)	0.484	0.094	0.067	0.273	0.24	0.353	
	*N*	135	135	135	135	135	135	135

** Correlation is significant at the 0.01 level (two-tailed).

**Table 3:** Correlation of MN1, MEIS1, MEIS2 and MEIS3 gene expression in patients with AML with other karyotypes

*Pearson correlations*								
		*MN1*	*MEIS1_p1*	*MEIS1_p2*	*MEIS1_p3*	*MEIS2*	*MEIS3_p1*	*MEIS3_p2*
MN1	Pearson correlation	1	0.033	0.033	−0.044	0.035	0.104	−0.139
	Sig. (two-tailed)			0.974	0.753	0.676	0.739	0.188
	*N*	91	91	91	91	91	91	91
MEIS1_p1	Pearson correlation	0.003	1	0.980**	0.940**	−0.269**	−0.011	−0.141
	Sig. (two-tailed)	0.974		0	0	0.01	0.914	0.184
	*N*	91	91	91	91	91	91	91
MEIS1_p2	Pearson correlation	0.033	0.980**	1	0.922**	−0.287**	0.019	−0.135
	Sig. (two-tailed)	0.753	0		0	0.006	0.86	0.203
	*N*	91	91	91	91	91	91	91
MEIS1_p3	Pearson correlation	−0.044	0.940**	0.922**	1	−0.210*	−0.016	−0.116
	Sig. (two-tailed)	0.676	0	0		0.046	0.878	0.273
	*N*	91	91	91	91	91	91	91
MEIS2	Pearson correlation	0.035	−0.269**	−0.287**	−0.210*	1	−0.021	−0.031
	Sig. (two-tailed)	0.739	0.01	0.006	0.046		0.845	0.771
	*N*	91	91	91	91	91	91	91
MEIS3_p1	Pearson correlation	0.104	−0.011	0.019	−0.016	−0.021	1	−0.115
	Sig. (two-tailed)	0.325	0.914	0.86	0.878	0.845		0.279
	*N*	91	91	91	91	91	91	91
MEIS3_p2	Pearson correlation	−0.139	−0.141	−0.135	−0.116	−0.031	−0.115	1
	Sig. (two-tailed)	0.188	0.184	0.203	0.273	0.771	0.279	
	*N*	91	91	91	91	91	91	91

* Correlation is significant at the 0.05 level (two-tailed).

** Correlation is significant at the 0.01 level (two-tailed).

## References

[bib1] Liu PP, Hajra A, Wijmenga C, Collins FS. Molecular pathogenesis of the chromosome 16 inversion in the M4Eo subtype of acute myeloid leukemia. Blood 1995; 85: 2289–2302. 7727763

[bib2] Valk PJ, Verhaak RG, Beijen MA, Erpelinck CA, Barjesteh van Waalwijk van Doorn-Khosrovani S, Boer JM et al. Prognostically useful gene-expression profiles in acute myeloid leukemia. N Engl J Med 2004; 350: 1617–1628. 15084694 10.1056/NEJMoa040465

[bib3] Heuser M, Beutel G, Krauter J, Dohner K, von Neuhoff N, Schlegelberger B et al. High meningioma 1 (MN1) expression as a predictor for poor outcome in acute myeloid leukemia with normal cytogenetics. Blood 2006; 108: 3898–3905. 16912223 10.1182/blood-2006-04-014845

[bib4] Langer C, Marcucci G, Holland KB, Radmacher MD, Maharry K, Paschka P et al. Prognostic importance of MN1 transcript levels, and biologic insights from MN1-associated gene and microRNA expression signatures in cytogenetically normal acute myeloid leukemia: a cancer and leukemia group B study. J Clin Oncol 2009; 27: 3198–3204. 19451432 10.1200/JCO.2008.20.6110 PMC2716941

[bib5] Metzeler KH, Dufour A, Benthaus T, Hummel M, Sauerland MC, Heinecke A et al. ERG expression is an independent prognostic factor and allows refined risk stratification in cytogenetically normal acute myeloid leukemia: a comprehensive analysis of ERG, MN1, and BAALC transcript levels using oligonucleotide microarrays. J Clin Oncol 2009; 27: 5031–5038. 19752345 10.1200/JCO.2008.20.5328

[bib6] Kandilci A, Grosveld GC. Reintroduction of CEBPA in MN1-overexpressing hematopoietic cells prevents their hyperproliferation and restores myeloid differentiation. Blood 2009; 114: 1596–1606. 19561324 10.1182/blood-2009-02-205443 PMC2731639

[bib7] Carella C, Bonten J, Sirma S, Kranenburg TA, Terranova S, Klein-Geltink R et al. MN1 overexpression is an important step in the development of inv(16) AML. Leukemia 2007; 21: 1679–1690. 17525718 10.1038/sj.leu.2404778

[bib8] Heuser M, Argiropoulos B, Kuchenbauer F, Yung E, Piper J, Fung S et al. MN1 overexpression induces acute myeloid leukemia in mice and predicts ATRA resistance in patients with AML. Blood 2007; 110: 1639–1647. 17494859 10.1182/blood-2007-03-080523

[bib9] Liu T, Jankovic D, Brault L, Ehret S, Baty F, Stavropoulou V et al. Functional characterization of high levels of meningioma 1 as collaborating oncogene in acute leukemia. Leukemia 2010; 24: 601–612. 20072157 10.1038/leu.2009.272

[bib10] Heuser M, Yun H, Berg T, Yung E, Argiropoulos B, Kuchenbauer F et al. Cell of origin in AML: susceptibility to MN1-induced transformation is regulated by the MEIS1/AbdB-like HOX protein complex. Cancer Cell 2011; 20: 39–52. 21741595 10.1016/j.ccr.2011.06.020 PMC3951989

[bib11] Vegi NM, Klappacher J, Oswald F, Mulaw MA, Mandoli A, Thiel VN et al. MEIS2 is an oncogenic partner in AML1-ETO-positive AML. Cell Rep 2016; 16: 498–507. 27346355 10.1016/j.celrep.2016.05.094

[bib12] Fellmann C, Hoffmann T, Sridhar V, Hopfgartner B, Muhar M, Roth M et al. An optimized microRNA backbone for effective single-copy RNAi. Cell Rep 2013; 5: 1704–1713. 24332856 10.1016/j.celrep.2013.11.020

[bib13] Maetzig T, Ruschmann J, Lai CK, Ngom M, Imren S, Rosten P et al. A lentiviral fluorescent genetic barcoding system for flow cytometry-based multiplex tracking. Mol Ther 2017; 25: 606–620. 28253481 10.1016/j.ymthe.2016.12.005 PMC5363216

[bib14] Lai CK, Moon Y, Kuchenbauer F, Starzcynowski DT, Argiropoulos B, Yung E et al. Cell fate decisions in malignant hematopoiesis: leukemia phenotype is determined by distinct functional domains of the MN1 oncogene. PloS One 2014; 9: e112671. 25401736 10.1371/journal.pone.0112671 PMC4234417

[bib15] Imren S, Heuser M, Gasparetto M, Beer PA, Norddahl GL, Xiang P et al. Modeling de novo leukemogenesis from human cord blood with MN1 and NUP98HOXD13. Blood 2014; 124: 3608–3612. 25339361 10.1182/blood-2014-04-564666 PMC4256911

[bib16] Miller ME, Rosten P, Lemieux ME, Lai C, Humphries RK. Meis1 is required for adult mouse erythropoiesis, megakaryopoiesis and hematopoietic stem cell expansion. PloS One 2016; 11: e0151584. 26986211 10.1371/journal.pone.0151584 PMC4795694

[bib17] Subramanian A, Tamayo P, Mootha VK, Mukherjee S, Ebert BL, Gillette MA et al. Gene set enrichment analysis: a knowledge-based approach for interpreting genome-wide expression profiles. Proc Natl Acad Sci USA 2005; 102: 15545–15550. 16199517 10.1073/pnas.0506580102 PMC1239896

[bib18] Hulsen T, de Vlieg J, Alkema W. BioVenn—a web application for the comparison and visualization of biological lists using area-proportional Venn diagrams. BMC Genomics 2008; 9: 488. 18925949 10.1186/1471-2164-9-488 PMC2584113

[bib19] Patro R, Mount SM, Kingsford C. Sailfish enables alignment-free isoform quantification from RNA-seq reads using lightweight algorithms. Nat Biotechnol 2014; 32: 462–464. 24752080 10.1038/nbt.2862 PMC4077321

[bib20] Koboldt DC, Zhang Q, Larson DE, Shen D, McLellan MD, Lin L et al. VarScan 2: somatic mutation and copy number alteration discovery in cancer by exome sequencing. Genome Res 2012; 22: 568–576. 22300766 10.1101/gr.129684.111 PMC3290792

[bib21] Benjamini Y, Hochberg Y. Controlling the false discovery rate: a practical and powerful approach to multiple testing. J R Stat Soc Ser B 1995; 57: 289–300.

[bib22] Team RCR: A language and environment for statistical computing. R Foundation for Statistical Computing Vienna: Austria, 2016, Available from https://www.R-project.org/ (accessed 19 December 2016).

[bib23] Lemieux S, Sargeant T, Laperriere D, Ismail H, Boucher G, Rozendaal M et al. MiSTIC, an integrated platform for the analysis of heterogeneity in large tumour transcriptome datasets. Nucleic Acids Res 2017; e-pub ahead of print 27 july 2017; doi: 10.1093/nar/gkx338. 10.1093/nar/gkx338 PMC5570030 28472340

[bib24] Bagger FO, Sasivarevic D, Sohi SH, Laursen LG, Pundhir S, Sonderby CK et al. BloodSpot: a database of gene expression profiles and transcriptional programs for healthy and malignant haematopoiesis. Nucleic Acids Res 2016; 44: D917–D924. 26507857 10.1093/nar/gkv1101 PMC4702803

[bib25] Sharma A, Yun H, Jyotsana N, Chaturvedi A, Schwarzer A, Yung E et al. Constitutive IRF8 expression inhibits AML by activation of repressed immune response signaling. Leukemia 2015; 29: 157–168. 24957708 10.1038/leu.2014.162

[bib26] Lapidot T, Sirard C, Vormoor J, Murdoch B, Hoang T, Caceres-Cortes J et al. A cell initiating human acute myeloid leukaemia after transplantation into SCID mice. Nature 1994; 367: 645–648. 7509044 10.1038/367645a0

[bib27] Bonnet D, Dick JE. Human acute myeloid leukemia is organized as a hierarchy that originates from a primitive hematopoietic cell. Nat Med 1997; 3: 730–737. 9212098 10.1038/nm0797-730

[bib28] Eppert K, Takenaka K, Lechman ER, Waldron L, Nilsson B, van Galen P et al. Stem cell gene expression programs influence clinical outcome in human leukemia. Nat Med 2011; 17: 1086–1093. 21873988 10.1038/nm.2415

[bib29] Goardon N, Marchi E, Atzberger A, Quek L, Schuh A, Soneji S et al. Coexistence of LMPP-like and GMP-like leukemia stem cells in acute myeloid leukemia. Cancer Cell 2011; 19: 138–152. 21251617 10.1016/j.ccr.2010.12.012

[bib30] Horton SJ, Jaques J, Woolthuis C, van Dijk J, Mesuraca M, Huls G et al. MLL-AF9-mediated immortalization of human hematopoietic cells along different lineages changes during ontogeny. Leukemia 2013; 27: 1116–1126. 23178754 10.1038/leu.2012.343

[bib31] Kawagoe H, Humphries RK, Blair A, Sutherland HJ, Hogge DE. Expression of HOX genes, HOX cofactors, and MLL in phenotypically and functionally defined subpopulations of leukemic and normal human hematopoietic cells. Leukemia 1999; 13: 687–698. 10374871 10.1038/sj.leu.2401410

[bib32] Rozovskaia T, Feinstein E, Mor O, Foa R, Blechman J, Nakamura T et al. Upregulation of Meis1 and HoxA9 in acute lymphocytic leukemias with the t(4: 11) abnormality. Oncogene 2001; 20: 874–878. 11314021 10.1038/sj.onc.1204174

[bib33] Imamura T, Morimoto A, Takanashi M, Hibi S, Sugimoto T, Ishii E et al. Frequent co-expression of HoxA9 and Meis1 genes in infant acute lymphoblastic leukaemia with MLL rearrangement. Br J Haematol 2002; 119: 119–121. 12358913 10.1046/j.1365-2141.2002.03803.x

[bib34] Grubach L, Juhl-Christensen C, Rethmeier A, Olesen LH, Aggerholm A, Hokland P et al. Gene expression profiling of Polycomb, Hox and Meis genes in patients with acute myeloid leukaemia. Eur J Haematol 2008; 81: 112–122. 18410541 10.1111/j.1600-0609.2008.01083.x

[bib35] Pabst C, Bergeron A, Lavallee VP, Yeh J, Gendron P, Norddahl GL et al. GPR56 identifies primary human acute myeloid leukemia cells with high repopulating potential *in vivo*. Blood 2016; 127: 2018–2027. 26834243 10.1182/blood-2015-11-683649

[bib36] Zha Y, Xia Y, Ding J, Choi JH, Yang L, Dong Z et al. MEIS2 is essential for neuroblastoma cell survival and proliferation by transcriptional control of M-phase progression. Cell Death Dis 2014; 5: e1417. 25210800 10.1038/cddis.2014.370 PMC4540202

[bib37] Bjerke GA, Hyman-Walsh C, Wotton D. Cooperative transcriptional activation by Klf4, Meis2, and Pbx1. Mol Cell Biol 2011; 31: 3723–3733. 21746878 10.1128/MCB.01456-10 PMC3165729

[bib38] Schwind S, Marcucci G, Kohlschmidt J, Radmacher MD, Mrozek K, Maharry K et al. Low expression of MN1 associates with better treatment response in older patients with de novo cytogenetically normal acute myeloid leukemia. Blood 2011; 118: 4188–4198. 21828125 10.1182/blood-2011-06-357764 PMC3291490

[bib39] Heuser M, Sly LM, Argiropoulos B, Kuchenbauer F, Lai C, Weng A et al. Modeling the functional heterogeneity of leukemia stem cells: role of STAT5 in leukemia stem cell self-renewal. Blood 2009; 114: 3983–3993. 19667399 10.1182/blood-2009-06-227603

[bib40] Kroon E, Krosl J, Thorsteinsdottir U, Baban S, Buchberg AM, Sauvageau G. Hoxa9 transforms primary bone marrow cells through specific collaboration with Meis1a but not Pbx1b. EMBO J 1998; 17: 3714–3725. 9649441 10.1093/emboj/17.13.3714 PMC1170707

[bib41] Golub TR, Slonim DK, Tamayo P, Huard C, Gaasenbeek M, Mesirov JP et al. Molecular classification of cancer: class discovery and class prediction by gene expression monitoring. Science 1999; 286: 531–537. 10521349 10.1126/science.286.5439.531

[bib42] Thorsteinsdottir U, Kroon E, Jerome L, Blasi F, Sauvageau G. Defining roles for HOX and MEIS1 genes in induction of acute myeloid leukemia. Mol Cell Biol 2001; 21: 224–234. 11113197 10.1128/MCB.21.1.224-234.2001 PMC88796

[bib43] Thorsteinsdottir U, Mamo A, Kroon E, Jerome L, Bijl J, Lawrence HJ et al. Overexpression of the myeloid leukemia-associated Hoxa9 gene in bone marrow cells induces stem cell expansion. Blood 2002; 99: 121–129. 11756161 10.1182/blood.v99.1.121

[bib44] Ayton PM, Cleary ML. Transformation of myeloid progenitors by MLL oncoproteins is dependent on Hoxa7 and Hoxa9. Genes Dev 2003; 17: 2298–2307. 12952893 10.1101/gad.1111603 PMC196466

[bib45] Inaba T, Roberts WM, Shapiro LH, Jolly KW, Raimondi SC, Smith SD et al. Fusion of the leucine zipper gene HLF to the E2A gene in human acute B-lineage leukemia. Science 1992; 257: 531–534. 1386162 10.1126/science.1386162

[bib46] Shojaei F, Trowbridge J, Gallacher L, Yuefei L, Goodale D, Karanu F et al. Hierarchical and ontogenic positions serve to define the molecular basis of human hematopoietic stem cell behavior. Dev Cell 2005; 8: 651–663. 15866157 10.1016/j.devcel.2005.03.004

[bib47] Kato T, Sakata-Yanagimoto M, Nishikii H, Ueno M, Miyake Y, Yokoyama Y et al. Hes1 suppresses acute myeloid leukemia development through FLT3 repression. Leukemia 2015; 29: 576–585. 25234168 10.1038/leu.2014.281

[bib48] Wong P, Iwasaki M, Somervaille TC, So CW, Cleary ML. Meis1 is an essential and rate-limiting regulator of MLL leukemia stem cell potential. Genes Dev 2007; 21: 2762–2774. 17942707 10.1101/gad.1602107 PMC2045130

[bib49] Rosales-Avina JA, Torres-Flores J, Aguilar-Lemarroy A, Gurrola-Diaz C, Hernandez-Flores G, Ortiz-Lazareno PC et al. MEIS1, PREP1, and PBX4 are differentially expressed in acute lymphoblastic leukemia: association of MEIS1 expression with higher proliferation and chemotherapy resistance. J Exp Clin Cancer Res 2011; 30: 112. 22185299 10.1186/1756-9966-30-112 PMC3259065

[bib50] Mamo A, Krosl J, Kroon E, Bijl J, Thompson A, Mayotte N et al. Molecular dissection of Meis1 reveals 2 domains required for leukemia induction and a key role for Hoxa gene activation. Blood 2006; 108: 622–629. 16469876 10.1182/blood-2005-06-2244

[bib51] Heine P, Dohle E, Bumsted-O'Brien K, Engelkamp D, Schulte D. Evidence for an evolutionary conserved role of homothorax/Meis1/2 during vertebrate retina development. Development 2008; 135: 805–811. 18216174 10.1242/dev.012088

[bib52] Fujino T, Yamazaki Y, Largaespada DA, Jenkins NA, Copeland NG, Hirokawa K et al. Inhibition of myeloid differentiation by Hoxa9, Hoxb8, and Meis homeobox genes. Exp Hematol 2001; 29: 856–863. 11438208 10.1016/s0301-472x(01)00655-5

[bib53] Machon O, Masek J, Machonova O, Krauss S, Kozmik Z. Meis2 is essential for cranial and cardiac neural crest development. BMC Dev Biol 2015; 15: 40. 26545946 10.1186/s12861-015-0093-6 PMC4636814

[bib54] Oulad-Abdelghani M, Chazaud C, Bouillet P, Sapin V, Chambon P, Dolle P. Meis2, a novel mouse Pbx-related homeobox gene induced by retinoic acid during differentiation of P19 embryonal carcinoma cells. Dev Dynam 1997; 210: 173–183. 10.1002/(SICI)1097-0177(199710)210:2<173::AID-AJA9>3.0.CO;2-D 9337137

[bib55] Kocabas F, Zheng J, Thet S, Copeland NG, Jenkins NA, DeBerardinis RJ et al. Meis1 regulates the metabolic phenotype and oxidant defense of hematopoietic stem cells. Blood 2012; 120: 4963–4972. 22995899 10.1182/blood-2012-05-432260 PMC3525021

[bib56] Unnisa Z, Clark JP, Roychoudhury J, Thomas E, Tessarollo L, Copeland NG et al. Meis1 preserves hematopoietic stem cells in mice by limiting oxidative stress. Blood 2012; 120: 4973–4981. 23091297 10.1182/blood-2012-06-435800 PMC3525022

