# Supplementary material for: Meis2 as a critical player in MN1-induced leukemia
Source: Blood Cancer J. 2017 Sep 29;7(9):e613–. doi: 10.1038/bcj.2017.86 (PMC5709755; doi:10.1038/bcj.2017.86)
Supplement: Supplementary Information [file bcj201786x1.docx]

**Supplemental Data**

**Methods**

**Retroviral vectors, lentiviral shRNA vectors, and virus production**

The HA-MN1.IRES.GFP vector was previously described([1](#_ENREF_1)) and was used to generate retroviral vectors for MN1. Helper-free recombinant retrovirus was generated by using supernatants from the transfected ecotropic Phoenix packaging cell line to transduce the ecotropic GP+E86 packaging cell line([2](#_ENREF_2)).

Small hairpin RNA (shRNA) sequences were ordered as 97-mer([3](#_ENREF_3)) non-PAGE purified IDT Ultramers (Integrated DNA Technologies, Coralville, IA, USA). Ultramers were amplified with Platinum Taq DNA Polymerase (ThermoFisher Scientific, Carlsbad, CA, USA) to add *XhoI* and *EcoRI* restriction enzyme sites if necessary and subcloned into a third-generation pRRL.PPT.SF.meKO2.miR-30e.pre* lentiviral backbone equipped with the miR-E framework for enhanced shRNA activity([3](#_ENREF_3), [4](#_ENREF_4)). Alternatively, shRNA ultramers containing existing *XhoI* or *EcoRI* restriction enzyme recognition sites were amplified using Phusion DNA Polymerase (ThermoFisher Scientific, Carlsbad, CA, USA) and cloned directly into the pRRL.PPT.meKO2.miR30e.pre* lentiviral vector using Gibson Assembly Master Mix (New England Biolabs, Ipswich, MA, USA).

Lentiviruses were produced by seeding 7x10^6^ 293T cells per 10cm dish in Dulbecco’s modified Eagle’s medium with 4500 mg/L glucose (DMEM; StemCell Technologies Inc., Vancouver, Canada) supplemented with 0.1 mg/mL sodium pyruvate (ThermoFisher Scientific, Carlsbad, CA, USA), 100U/mL penicillin + 100 μg/mL streptomycin (PenStrep; ThermoFisher Scientific, Carlsbad, CA, USA), and 10% FBS Performance Plus (Gibco/ThermoFisher Scientific, Carlsbad, CA, USA). Sixteen hours after transfection, medium was replaced by DMEM supplemented with 0.1 mg/mL sodium pyruvate, PenStrep, 10% FBS Performance Plus, and 10mM HEPES (Gibco/ThermoFisher Scientific, Carlsbad, CA, USA). Vector supernatants were harvested 36 hours and 60 hours after transfection, filtered (0.22μM; Argos Technologies, IL, USA) and stored at -80^o^C.

**Lentiviral transduction of MN1 bone marrow cell lines**

Lentiviral transduction was performed by seeding 100,000 MN1 bone marrow cells per well in 96-well U-bottom plates (VWR/Falcon, Mississauga, Canada) in DMEM supplemented with 15% FBS(StemCell Technologies Inc., #6250, Vancouver, Canada), 10ng/mL hIL6, 6ng/mL mIL3, 100ng/mL mSCF (36SF medium), supplemented with 5μg/mL protamine sulfate (Sigma-Aldrich, Oakville, Canada) and adding 30μL unconcentrated viral supernatant for shRNAs of interest. After 24 hours, half of the media was removed from each well and the remaining contents were transferred to 48-well plates (Greiner Bio One, Fisher Scientific, Carlsbad, CA, USA) with additional 36SF medium. At 48 hours post-transduction, this process was repeated with remaining contents moved to a 6-well plate (VWR/Falcon). At 72 hours post-transduction, well contents were collected and prepared for flow cytometric sorting.

**Clonogenic progenitor assays**

Colony-forming cells (CFCs) were assayed in methylcellulose (MethoCult M3434 or MegaCult-C, Catalog No. 04964; STEMCELL Technologies, Vancouver, Canada). For each assay, freshly isolated and transduced unsorted bone marrow cells were plated in duplicate in Methocult medium (1000 cells/well). Colonies were evaluated microscopically seven days after plating using standard criteria.

**FACS analysis**

Cells were prepared for FACS analysis as previously described([5](#_ENREF_5)). Monoclonal antibodies used were phycoerythrin (PE)-labeled CD4 (clone H129.19) and CD8 (clone 53-6.7; both BD Biosciences, San Jose, CA, USA), allophycocyanin (APC)/Cy7-, PE/Cy7-, and APC-labeled c-Kit (CD117, clone 2B8), AF700-labeled Gr-1 (Ly6G/6C, clone RB6-8C5; all Biolegend, San Diego, CA, USA), PE/Cy7-labeled CD19 (clone 1D3), and APC-labelled CD11b (clone M1/70; both eBioscience, San Diego, CA, USA). Human cord blood AML ND13+MN1 cells [{Imren](https://d.docs.live.net/4f2dd8c9c3e7af9a/Thesis/Compiled%20Thesis/Thesis-170315.docx#_ENREF_126) et al. 2014} were sorted using PE-labeled G-protein receptor 56 (GPR56) (clone CG4; Biolegend, San Diego, CA, USA) and APC-labeled CD34 (STEMCELL Technologies, Vancouver, Canada).

For isolation of primary murine progenitor and mature cell populations, bone marrow was isolated and suspended in PBS supplemented with 2% FBS and red blood cells were lysed with PharmLyse reagent (BD Biosciences, San Jose, CA, USA) per manufacturer instructions. Cells were blocked for 20 minutes on ice in PBS supplemented with 5% rat sera (STEMCELL Technologies, Vancouver, Canada) and 1μg/1x10^6^ Fc receptor (FcR, CD16/32), then washed with PBS supplemented with 2% FBS. Cells to be sorted for GMPs, MEPs, or CMPs were stained directly without blocking. Antibodies used were as previously described([6](#_ENREF_6)).

Immunophenotypic analysis of murine cells was performed on stained cells filtered through a 45 μM filter (Argos Technologies, IL, USA) using a BD LSRFortessa (BD Biosciences, San Diego, CA, USA) in the presence of 1μM 4’,6-diamidino-2-phenylindole (DAPI, Sigma-Aldrich, Oakville, Canada).

**Bone marrow morphology**

Cytospin preparations were stained with Wright-Giemsa stain as previously described([5](#_ENREF_5)). Images were visualised using a Axioplan2 microscope (Zeiss, Oberkochen, Germany) and a 63x/1.4 numerical aperture objective and Nikon Immersion Oil (Nikon, Mississauga, Canada). Images were captured using OpenLab 5 (Improvision, Coventry, England).

**Supplemental Figures and Tables**


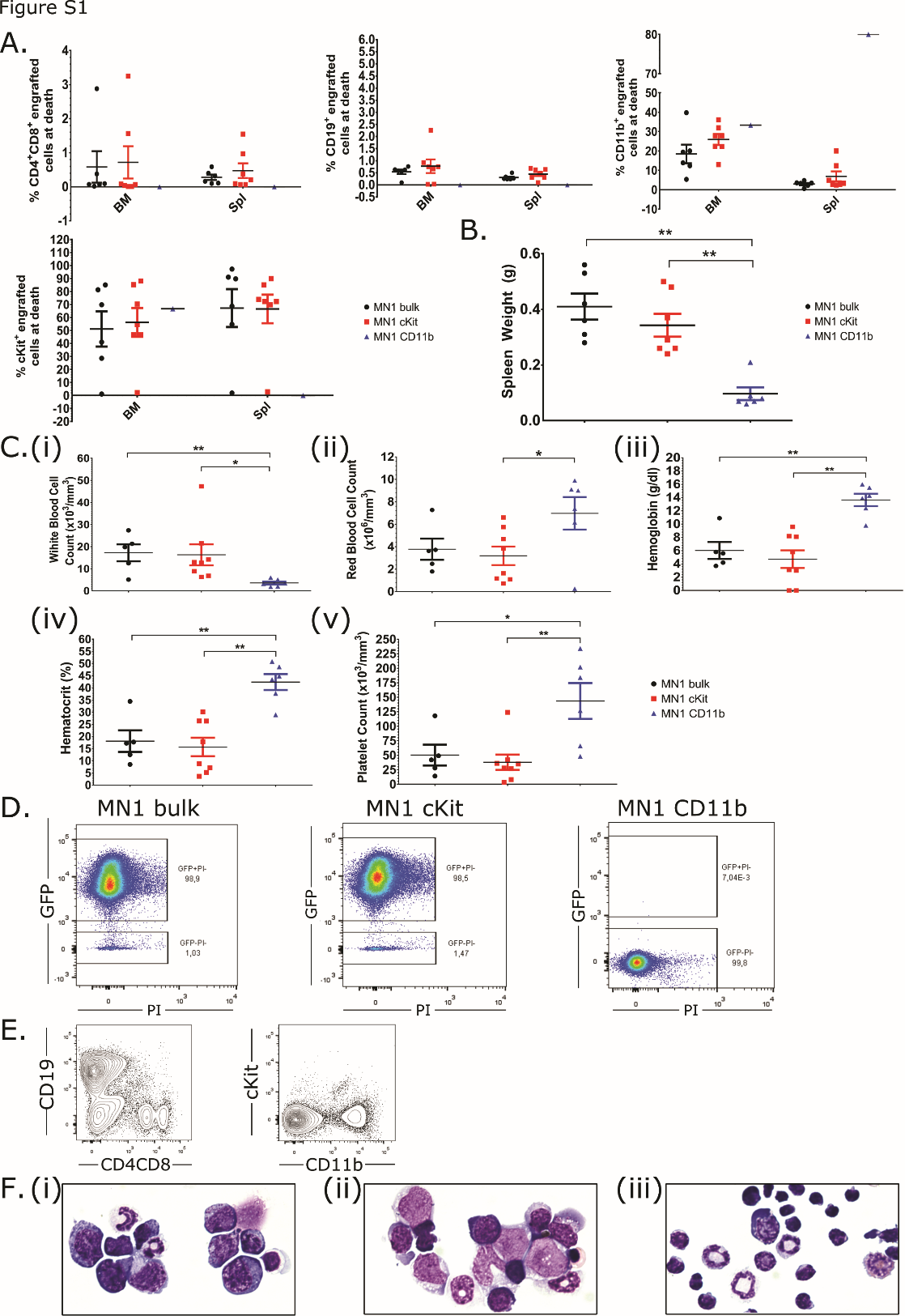


**Figure S1 Mice transplanted with CD11b cells are functionally devoid of leukemic initiating cell activity**

(A) Cell surface marker expression of engrafted CD4^+^CD8^+^, CD19^+^, CD11b^+^, and c-Kit^+^ cells in bone marrow in moribund/sacrificed secondary mice transplanted with MN1 bulk, cKit, and CD11b cells. n=6 for MN1 bulk, n=8 for cKit, and n=1 for CD11b. Unpaired two-sided t-test in MN1 bulk vs cKit/CD11b. Error bars represent ± SEM; *P<0.05, **P<0.01. (B) Mean spleen weight of mice transplanted with MN1 bulk, cKit, or CD11b cells isolated from leukemic MN1 mice at sacrifice. n=6 for MN1 bulk and CD11b cells, n=8 for cKit cells. Unpaired two-sided t-test in MN1 bulk vs cKit/CD11b. Error bars represent ± SEM; *P<0.05, P<0.01. (C)(i) White blood cell count, (ii) red blood cell count, (iii) hemoglobin measurement, (iv) percent hematocrit, and (v) platelet count in peripheral blood of moribund/sacrifice secondary mice transplanted with MN1 bulk, cKit, or CD11b cells. n=6 for MN1 bulk and CD11b cells, n=8 for cKit cells. Unpaired two-sided t-test in MN1 bulk vs cKit/CD11b. Error bars represent ±SEM; *P<0.05, **P<0.01. (D) Representative flow cytometric analysis on GFP^+^ bone marrow from mice transplanted with MN1 bulk, cKit, or CD11b cells. (E) Representative flow cytometric analysis on bone marrow from non-leukemic mouse transplanted with CD11b cells. (E) Representative cytospins of bone marrow from moribund/sacrificed mice transplanted with (i) MN1 bulk, (ii) cKit, and (iii) CD11b cells.


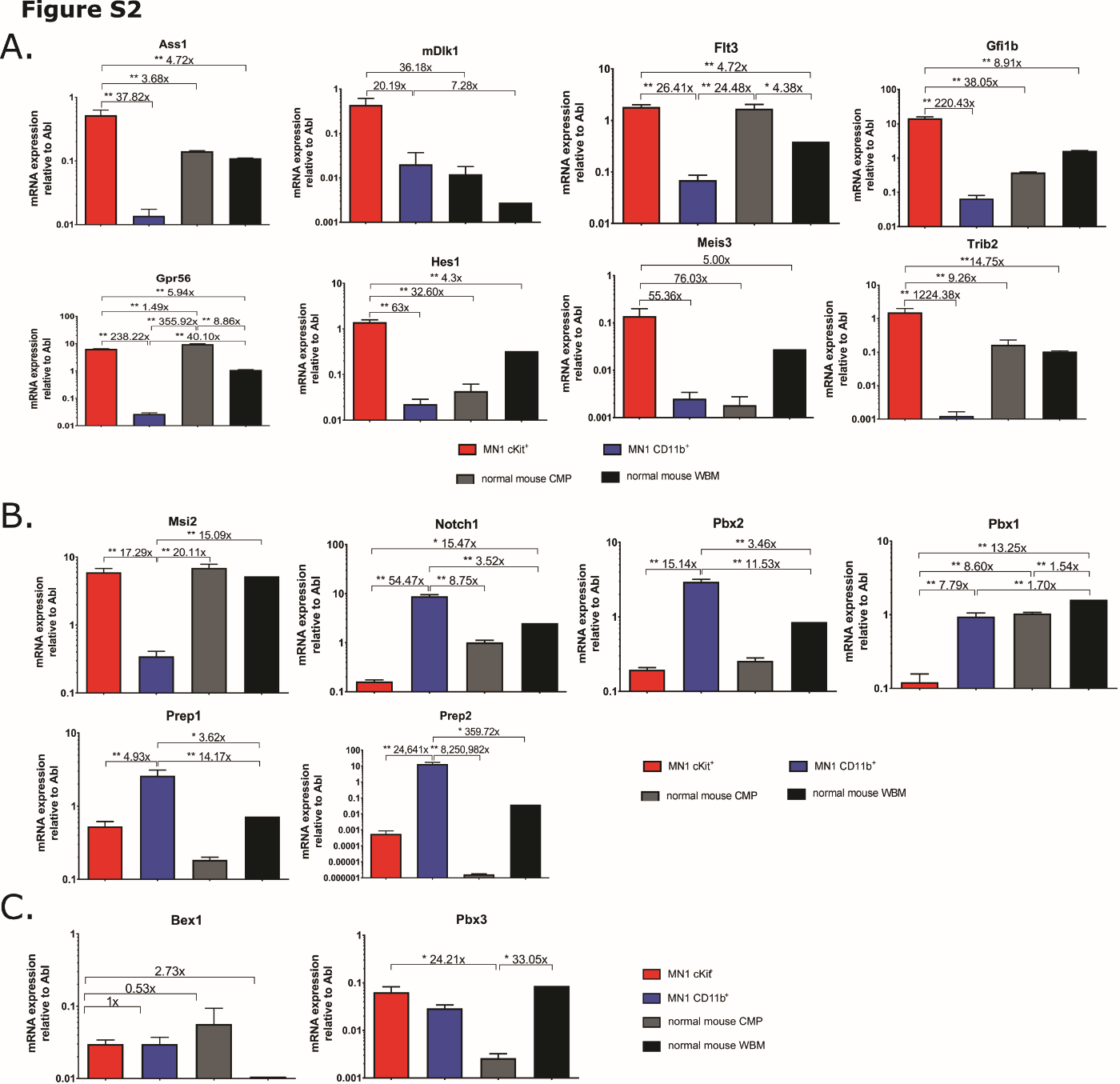


**Figure S2 Genes differentially expressed between multiple MN1 datasets modeling varying leukemic potencies reveal different patterns of expression**

(A) Absolute gene expression of candidate genes relative to Abl in cKit, CD11b, CMP, and whole bone marrow (WBM) cells by qRT-PCR, categorized by genes that are upregulated in cKit compared to CD11b cells, (C) genes that are upregulated in CD11b compared to cKit cells, and (D) genes that are expressed equally between cKit and CD11b cells, but are upregulated compared to gene expression levels in CMP and/or WBM. n=3 from four mice transplanted with cells from three independent transductions, one-sided ANOVA; error bars represent ±SEM; *P<0.05, **P<0.01.


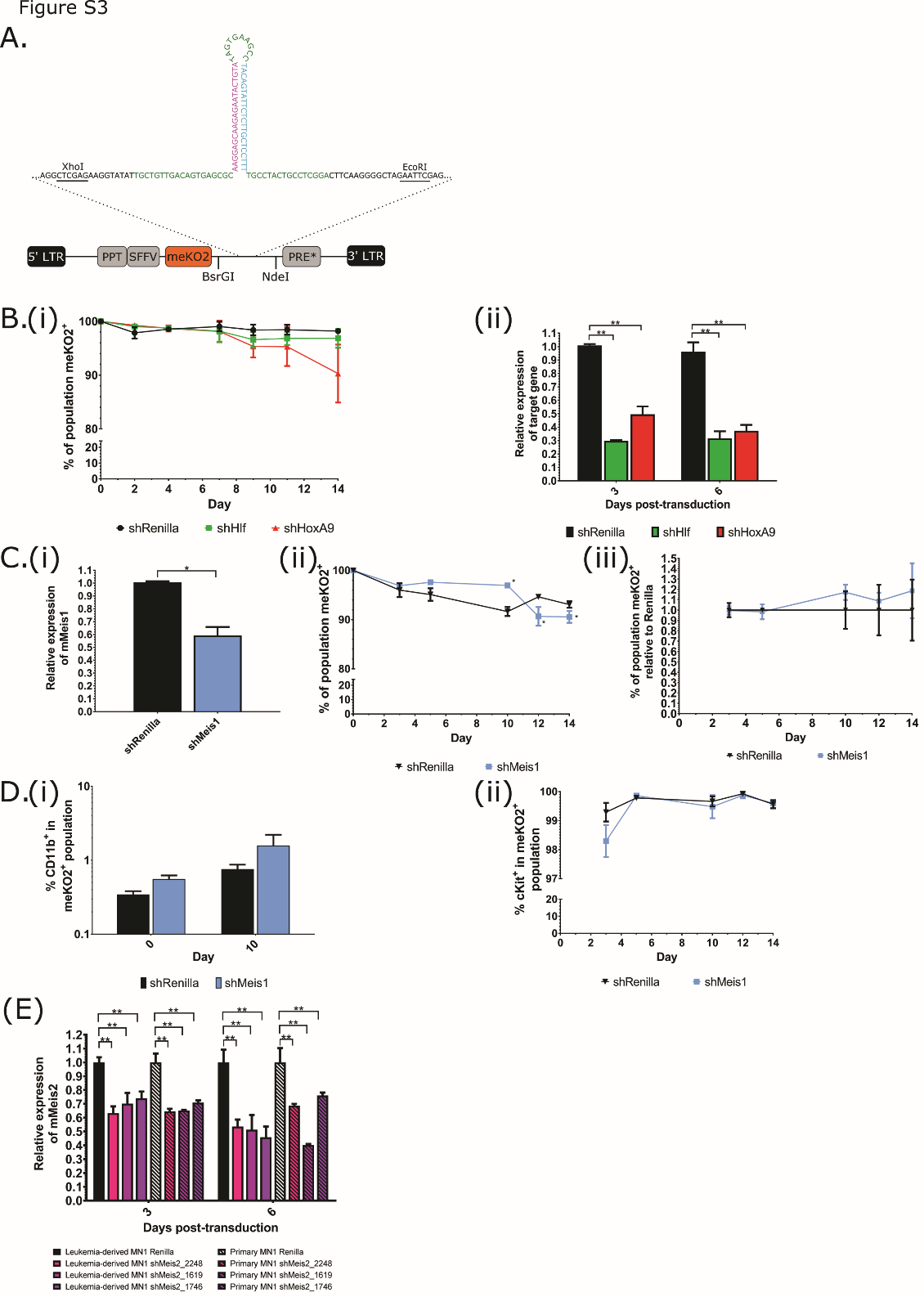


**Figure S3 shRNA lentiviral knockdown and functional validation**

(A) Schematic of shRNA lentiviral vector. Coloured sequence indicates 97-mer previously referenced([3](#_ENREF_3)). (magenta = guide sequence, blue = mRNA target sequence) Restriction enzyme recognition sequences are underlined and annotated. (B)(i) Relative mRNA expression of Hlf, HoxA9 in MN1 cells three and six days after shRNA transduction. (ii) (ii) Kinetics of meKO2^+^ expression of Renilla-, shHlf-, or shHoxa9-transduced MN1 cells after flow cytometric purification; n=3 from 2 independent experiments, unpaired t-test in Renilla vs shRNA; error bars represent ±SD; *P<0.05, **P<0.01. (C)(i) Relative mRNA expression of Meis1 in MN1 cells three days after shRNA transduction. (ii) Kinetics of meKO2^+^ expression of Renilla- and shMeis1-transduced MN1 cells after flow cytometric purification. (iii) Competitive growth assay containing mixed populations of 50% sorted untransduced MN1 cells and 50% sorted Renilla- or Meis1-transduced (meKO2^+^) MN1 cells. n=3 from 2 independent experiments, multiple two-sided t-test in Renilla vs shRNA; error bars represent ± SD; *P<0.05, **P<0.01. (D)(i) CD11b expression of Renilla- and shMeis1*-*transduced MN1 cell lines 10 days post-sort. (ii) Kinetics of c-Kit^+^ expression in Renilla- or shMeis1-transduced MN1 cells. Sorted meKO2^+^ cells; n=3 from 2 independent experiments, multiple two-sided t-test in Renilla vs shMeis1; error bars represent ± SD; *P<0.05, **P<0.01. (E) Relative mRNA expression of Meis2 in MN1 cells there and six days after shRNA transduction. Hlf, HoxA9 and Meis1: n=3 from 2 independent experiments, Meis2: n=4 from three (shMeis2_2248) or two (shMeis2_1619 and shMeis2_1746) independent experiments, unpaired t-test in Renilla vs shRNA; error bars represent ±SD; *P<0.05, **P<0.01.


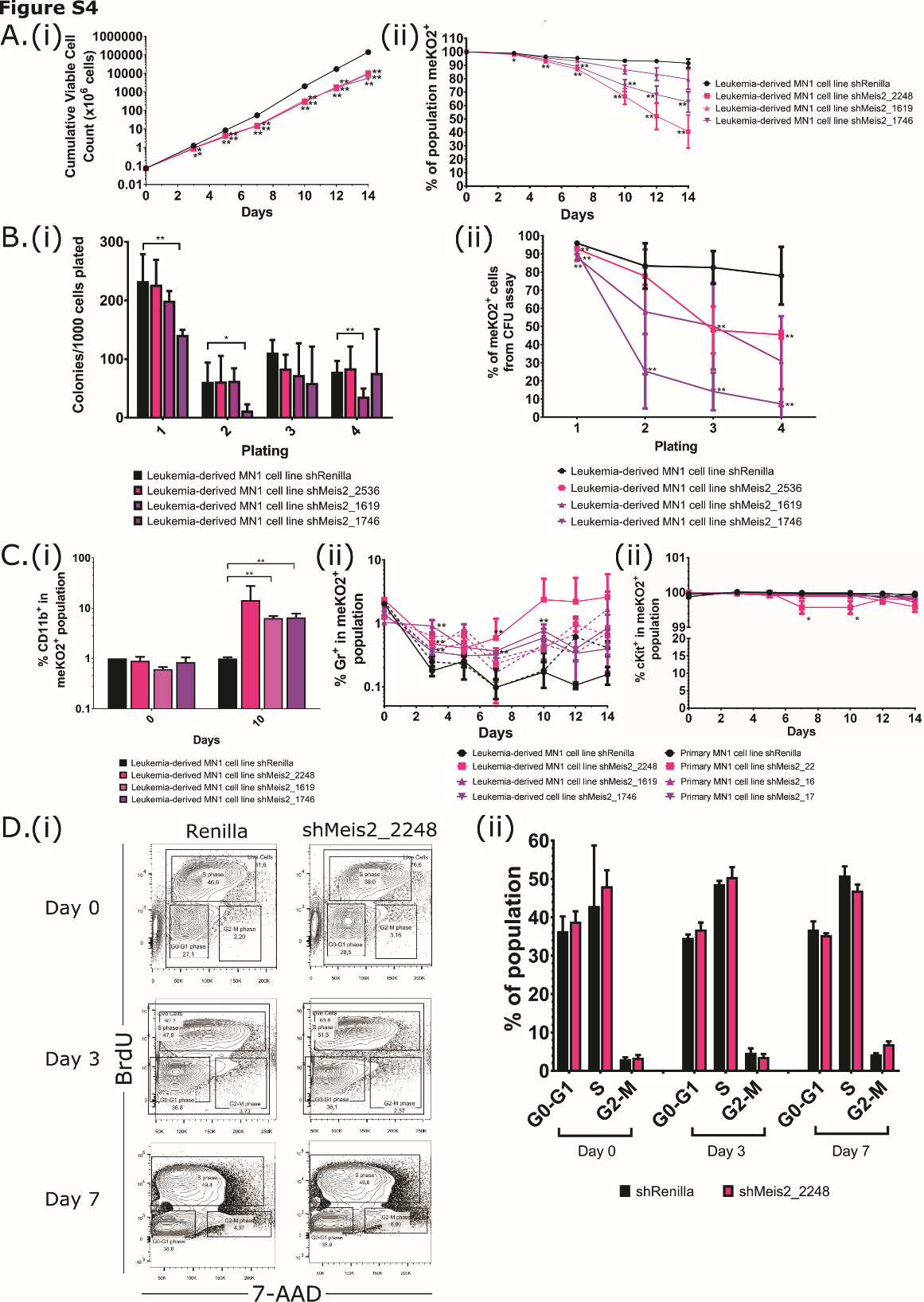


**Figure S4 Investigating the functional relevance of Meis2 on MN1 leukemic properties**

(A) Kinetics of (i) Gr-1^+^ and (ii) c-Kit^+^ expression in Renilla- and shMeis2-transduced MN1 cells. Sorted meKO2^+^ MN1 cells; n=3 from 3 (shMeis2_2248) or 2 (shMeis2_1619 and shMeis2_1746) independent experiments; error bars represent ± SEM; *P<0.05, **P<0.01. (B)(i) Representative cell cycle distribution (BrdU incorporation/7-aminoactinomycin D, 7-AAD) flow cytometric analysis in Renilla- and shMeis2-transduced *ex vivo*-derived MN1 cell line at day 0, 3, and 7 post-transduction. (ii) Summary of cell cycle distribution (BrdU incorporation/7-AAD) in Renilla- and shMeis2-transduced *ex vivo*-derived MN1 cell line. meKO2^+^ sorted cells, n=3 from 3 independent experiments. Error bars represent ± SEM; *P<0.05, **P<0.01.


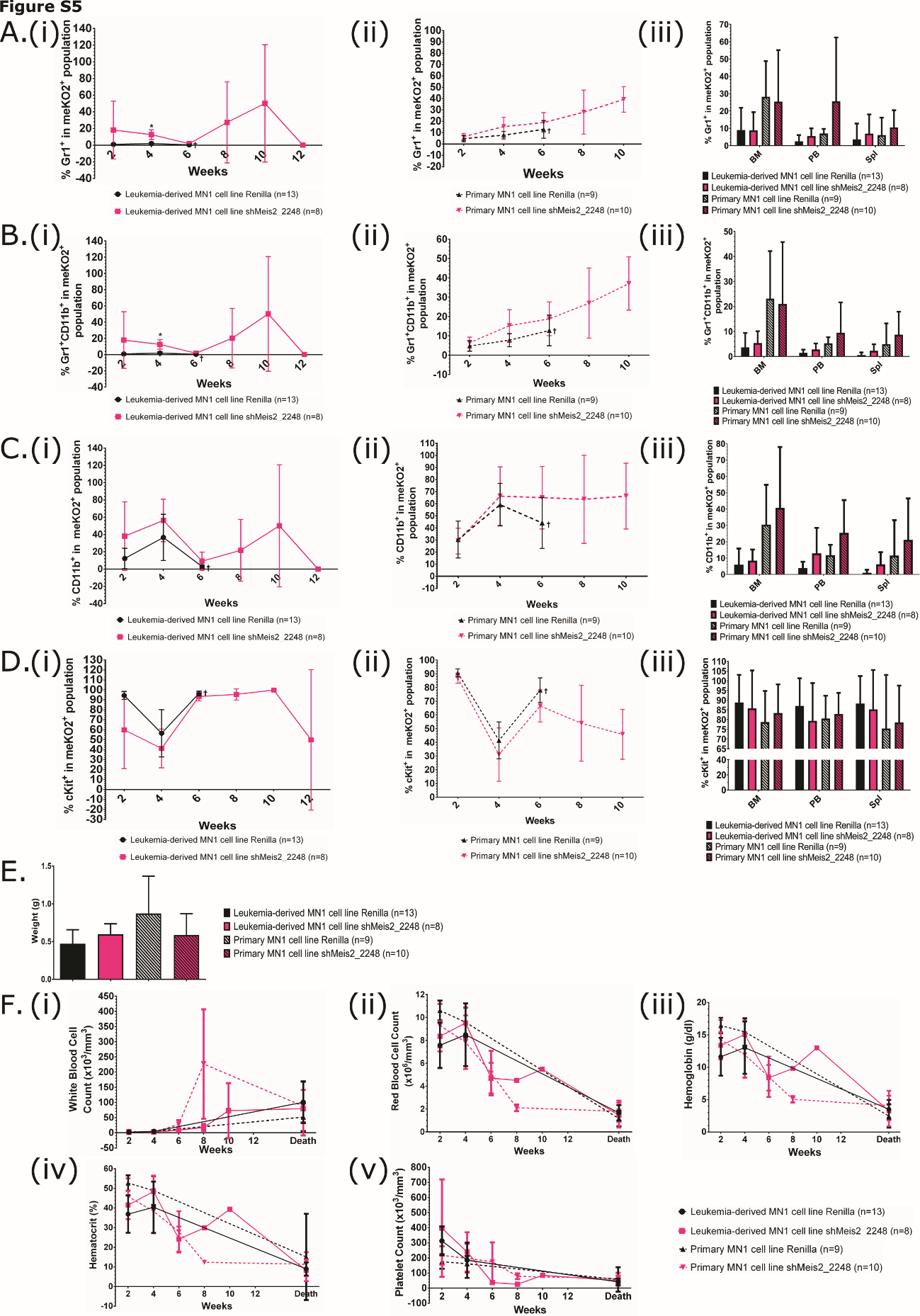


**Figure S5 Mice transplanted with shMeis2-transduced cells develop leukemia**

(A) Kinetics of Gr-1^+^ expression in meKO2^+^ engrafted bone marrow of mice transplanted with Renilla- or shMeis2-transduced (i) leukemia-derived- and (ii) primary MN1 cell lines and (iii) in bone marrow, peripheral blood, and spleen cells at sacrifice. (B) Kinetics of Gr-1^+^CD11b^+^ expression in meKO2^+^ engrafted bone marrow of mice transplanted with Renilla- or shMeis2-transduced (i) leukemia-derived and (ii) primary MN1 cell lines and (iii) in bone marrow, peripheral blood, and spleen cells at sacrifice. (C) Kinetics of CD11b^+^ expression in meKO2^+^ engrafted bone marrow of mice transplanted with Renilla- or shMeis2-transduced (i) leukemia-derived and (ii) primary MN1 cell lines and (iii) in bone marrow, peripheral blood, and spleen cells at sacrifice. (D) Kinetics of c-Kit^+^ expression in meKO2^+^ engrafted bone marrow of mice transplanted with Renilla- or shMeis2-transduced (i) leukemia-derived and (ii) primary MN1 cell lines and (iii) in bone marrow, peripheral blood, and spleen cells at sacrifice. (E) Mean spleen weight of mice transplanted with Renilla- or shMeis2-transduced leukemia-derived or primary MN1 cell lines at sacrifice. (F) Kinetics of (i) white blood cell count, (ii) red blood cell count, (iii) platelet count, (iv) hemoglobin concentration, and (v) hematocrit percentage in peripheral blood of mice transplanted with Renilla- or shMeis2-transduced leukemia-derived and primary MN1 cell lines. Leukemia-derived: n=13 for Renilla, n=8 for shMeis2; primary: n=9 for Renilla, n=10 for shMeis2, two-sided t-test in Renilla vs shMeis2. Error bars represent ± SD; † indicates all mice were sacrificed after this timepoint due to disease, *P<0.05.


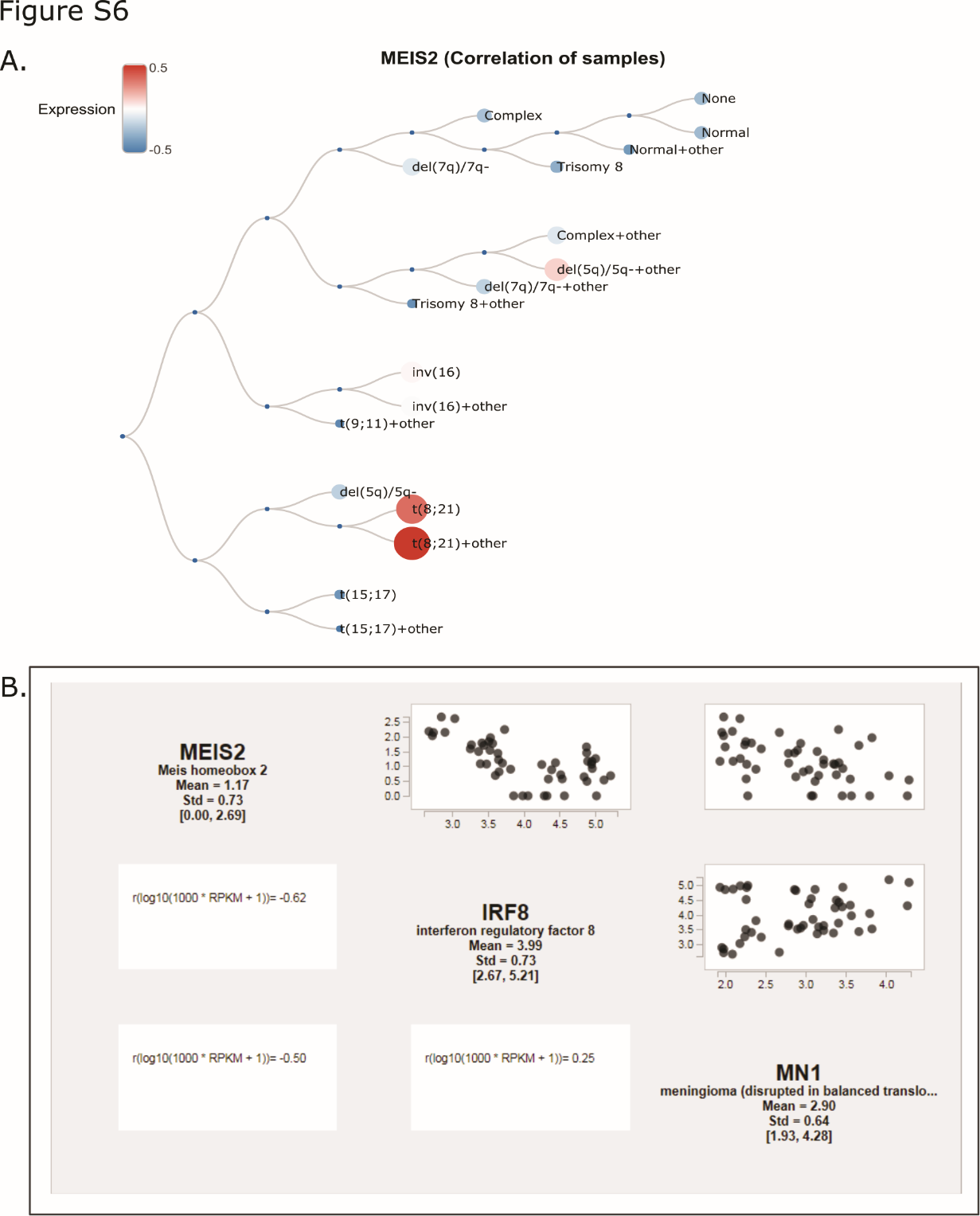


**Figure S6 MEIS2 expression from AML patient datasets**

(A) Hierarchical tree showing MESI2 expression levels in patients from TCGA dataset. (B) Pairwise correlation of MEIS2, IRF8, and MN1 expression from patients with normal karyotype AML Leucegene dataset. Mean, standard deviation (Std), and correlation (r) are shown.

Table S1 Primer Sequences for amplification of IDT Ultramers for cloning

| **Primer Name** | **Primer Sequence** |
| --- | --- |
| miRE-Xho-FWD | 5’-TGAACTCGAGAAGGTATATTGCTGTTGACAGTGAGCG-3’ |
| miRE-Eco-REV | 5’-TCTCGAATTCTAGCCCCTTGAAGTCCGAGGCAGTAGGC-3’ |
| MIR30e Lenti Gibson FWD | 5’-taacccaacagaaggctcgagAAGGTATATTGCTGTTGACAGTG-3’ |
| MIR30e Lenti Gibson REV | 5’- aaacaagataattgctcgaattcTAGCCCCTTGAAGTCCGA-3’ |

**References**

1. Heuser M, Yun H, Berg T, Yung E, Argiropoulos B, Kuchenbauer F, et al. Cell of origin in AML: susceptibility to MN1-induced transformation is regulated by the MEIS1/AbdB-like HOX protein complex. Cancer cell. 2011 Jul 12;20(1):39-52. PubMed PMID: 21741595. Pubmed Central PMCID: 3951989.

2. Gurevich RM, Aplan PD, Humphries RK. NUP98-topoisomerase I acute myeloid leukemia-associated fusion gene has potent leukemogenic activities independent of an engineered catalytic site mutation. Blood. 2004 Aug 15;104(4):1127-36. PubMed PMID: 15100157.

3. Fellmann C, Hoffmann T, Sridhar V, Hopfgartner B, Muhar M, Roth M, et al. An optimized microRNA backbone for effective single-copy RNAi. Cell reports. 2013 Dec 26;5(6):1704-13. PubMed PMID: 24332856.

4. Maetzig T, Ruschmann J, Lai CK, Ngom M, Imren S, Rosten P, et al. A Lentiviral Fluorescent Genetic Barcoding System for Flow Cytometry-Based Multiplex Tracking. Molecular therapy : the journal of the American Society of Gene Therapy. 2017 Mar 01;25(3):606-20. PubMed PMID: 28253481. Pubmed Central PMCID: 5363216.

5. Lai CK, Moon Y, Kuchenbauer F, Starzcynowski DT, Argiropoulos B, Yung E, et al. Cell fate decisions in malignant hematopoiesis: leukemia phenotype is determined by distinct functional domains of the MN1 oncogene. PloS one. 2014;9(11):e112671. PubMed PMID: 25401736. Pubmed Central PMCID: 4234417.

6. Miller ME, Rosten P, Lemieux ME, Lai C, Humphries RK. Meis1 Is Required for Adult Mouse Erythropoiesis, Megakaryopoiesis and Hematopoietic Stem Cell Expansion. PloS one. 2016;11(3):e0151584. PubMed PMID: 26986211. Pubmed Central PMCID: 4795694.
