# Supplementary figures and images for: Meis2 as a critical player in MN1-induced leukemia
Source: Blood Cancer J. 2017 Sep 29;7(9):e613–. doi: 10.1038/bcj.2017.86 (PMC5709755; doi:10.1038/bcj.2017.86)

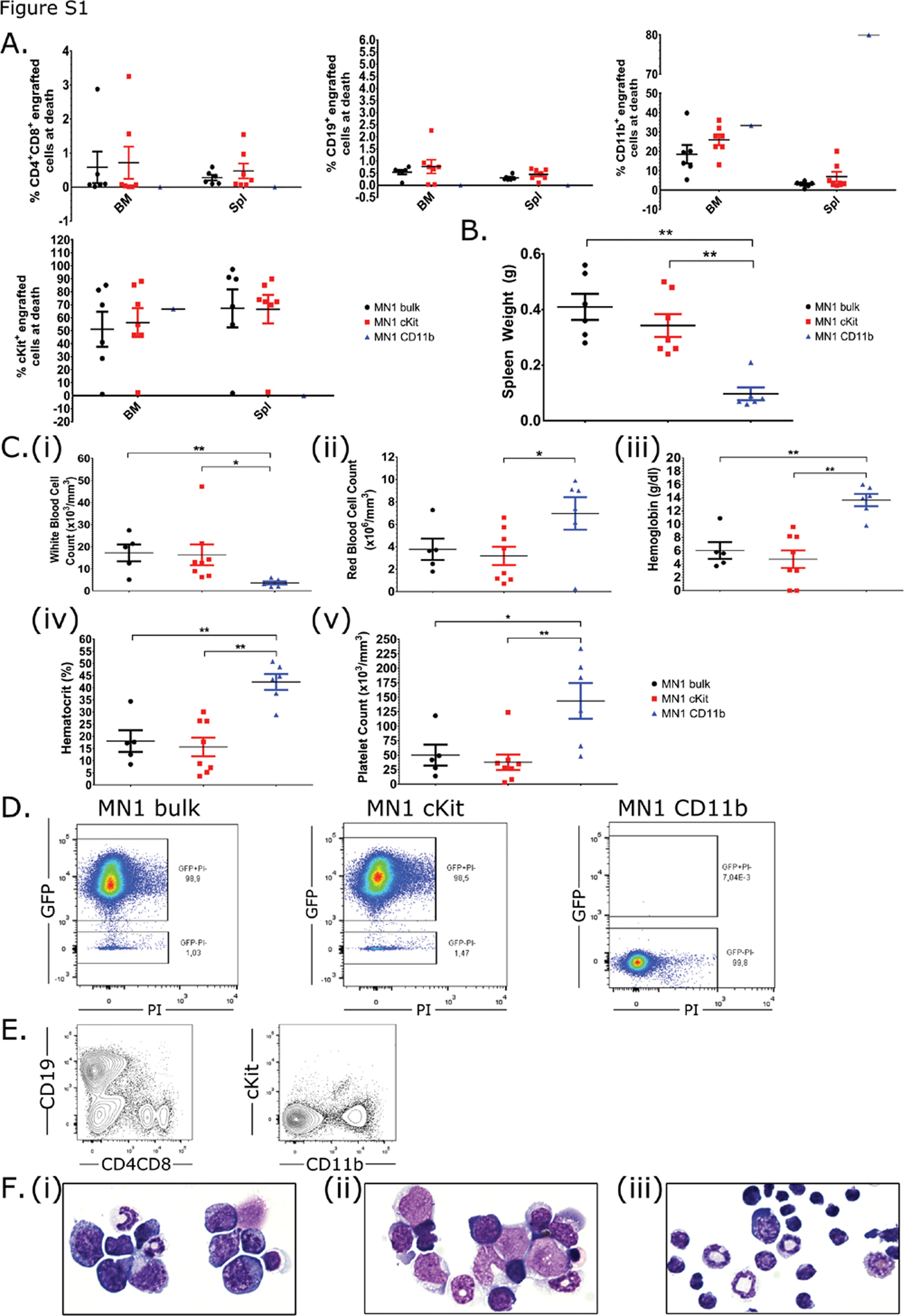

Supplement: Supplementary Figure S1 [file bcj201786x2.tif]

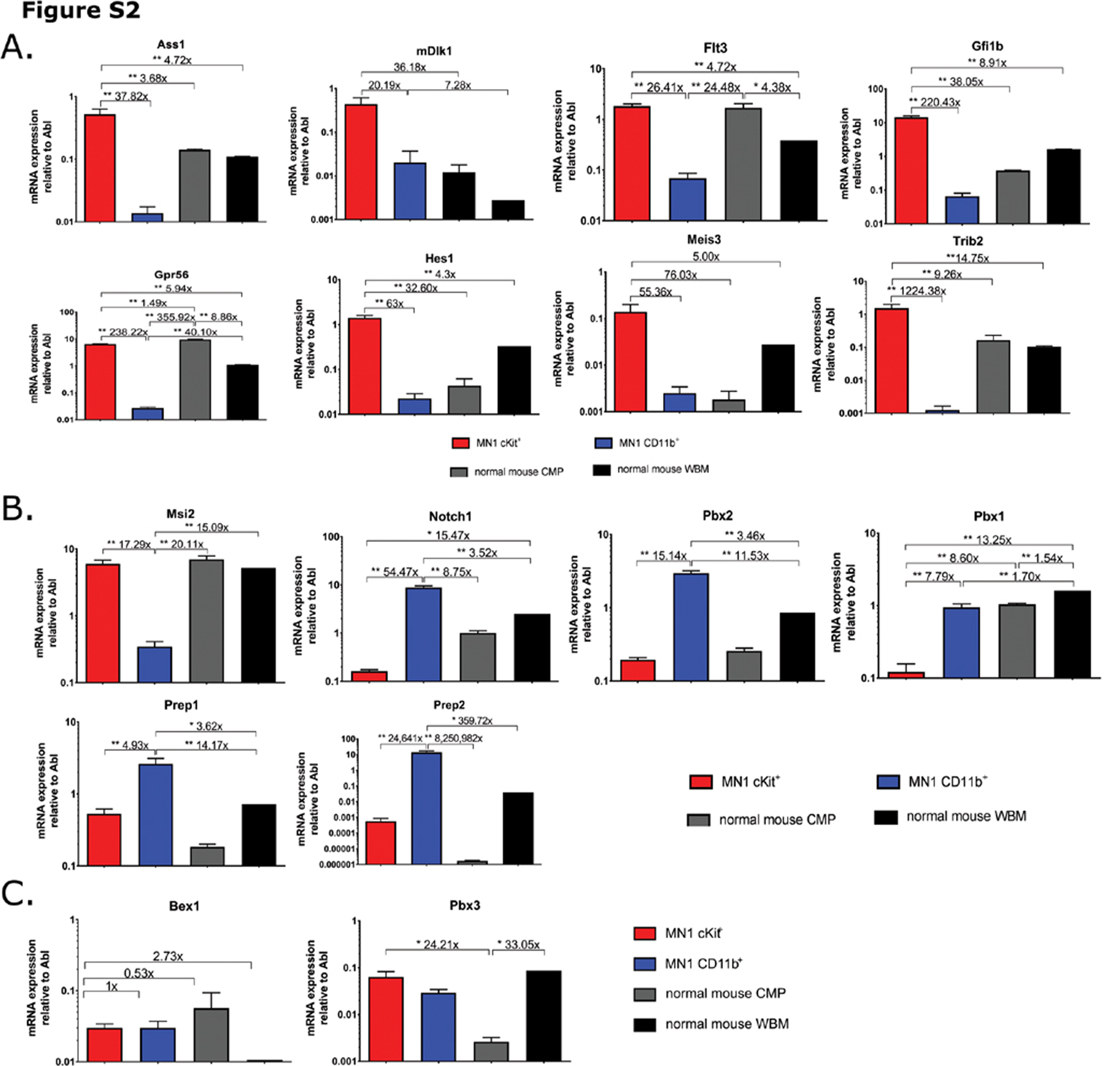

Supplement: Supplementary Figure S2 [file bcj201786x3.tif]

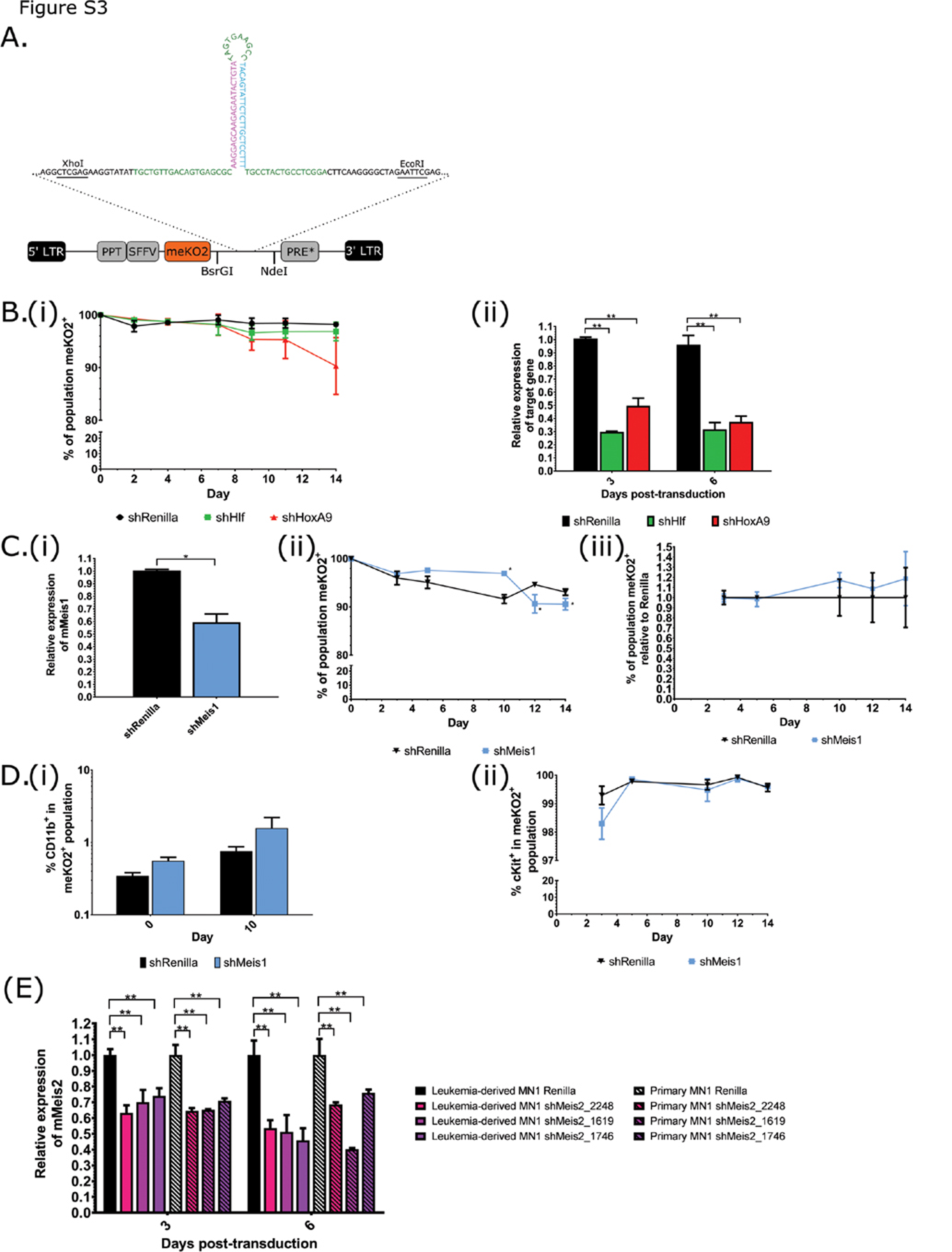

Supplement: Supplementary Figure S3 [file bcj201786x4.tif]

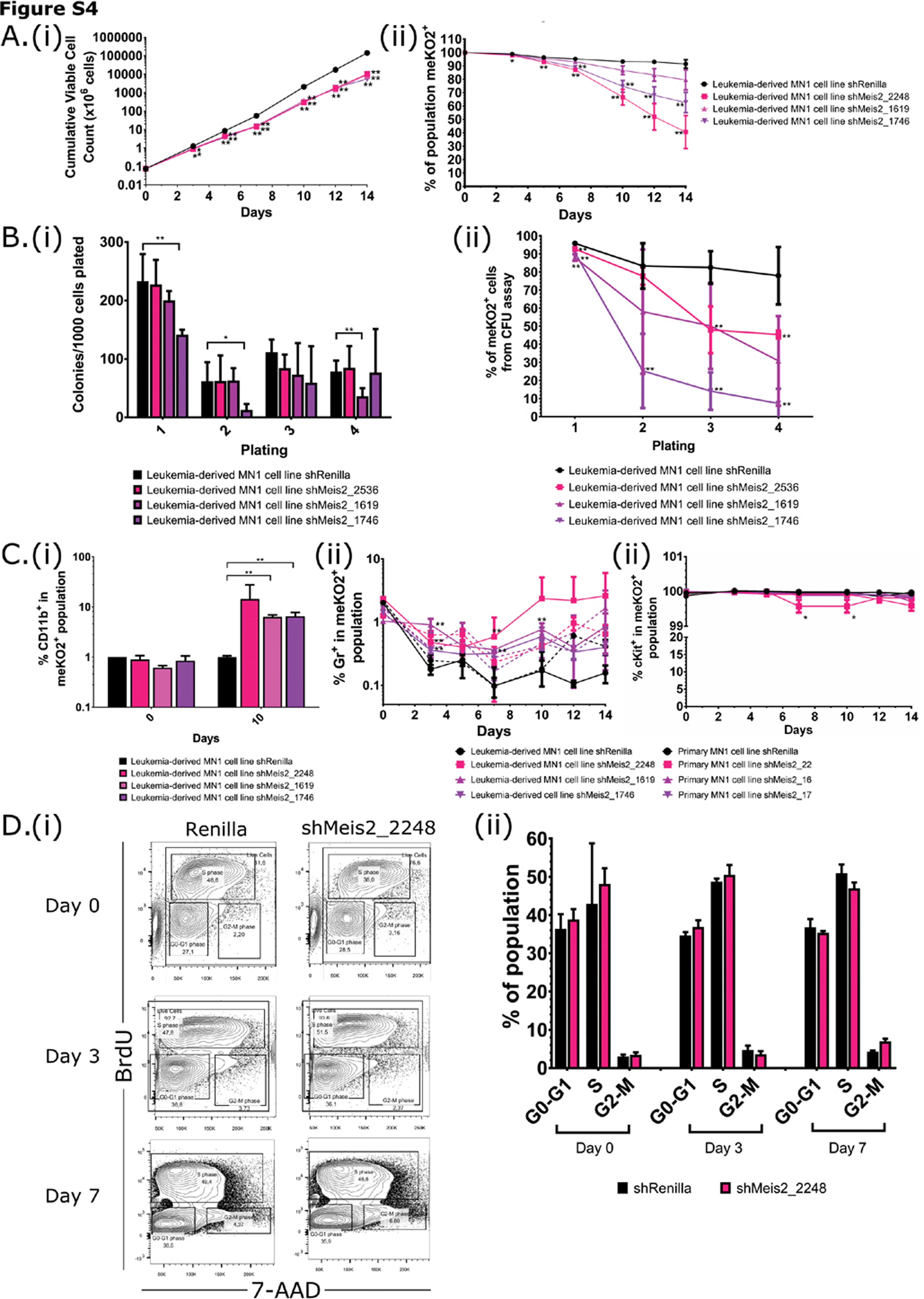

Supplement: Supplementary Figure S4 [file bcj201786x5.tif]

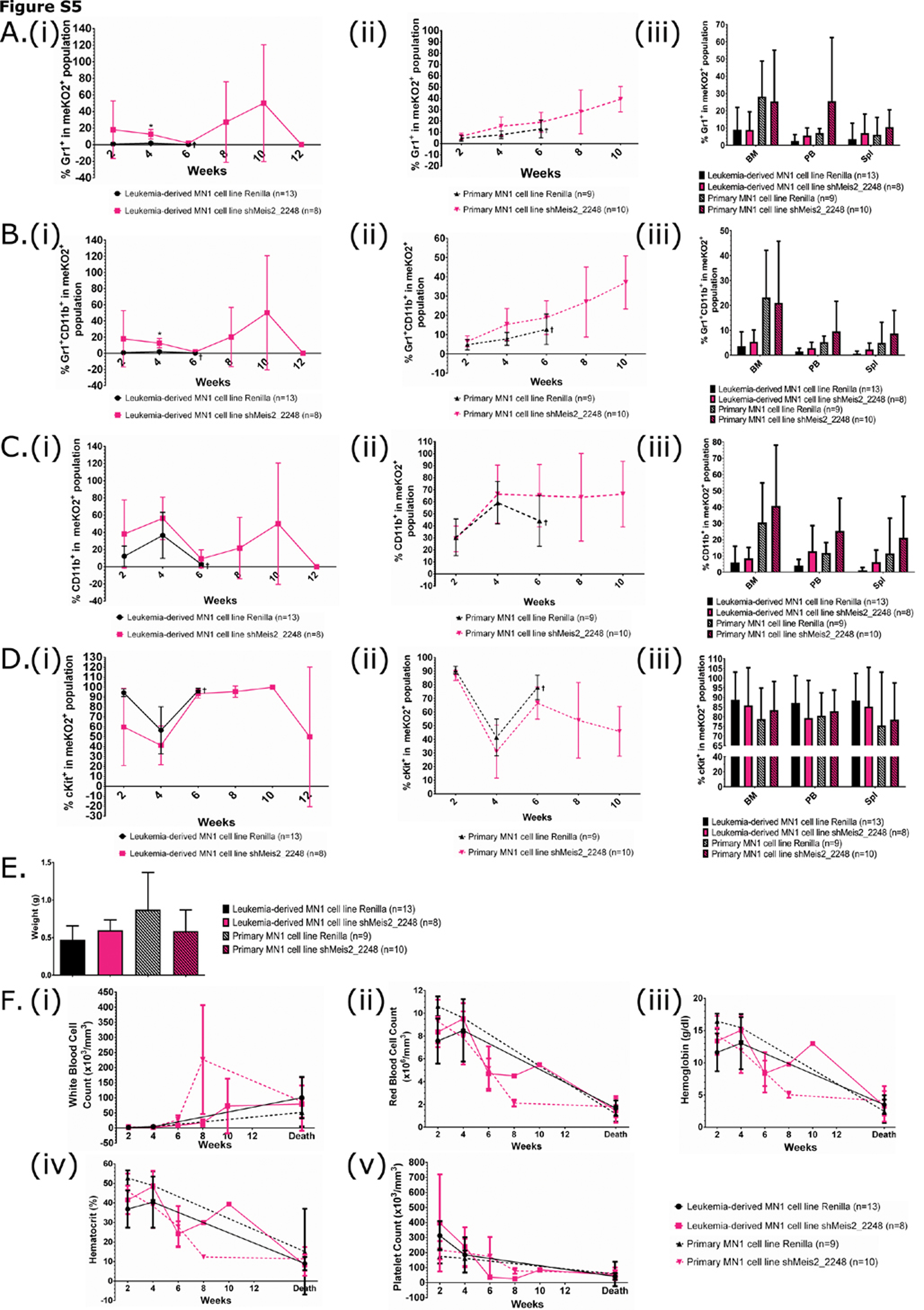

Supplement: Supplementary Figure S5 [file bcj201786x6.tif]

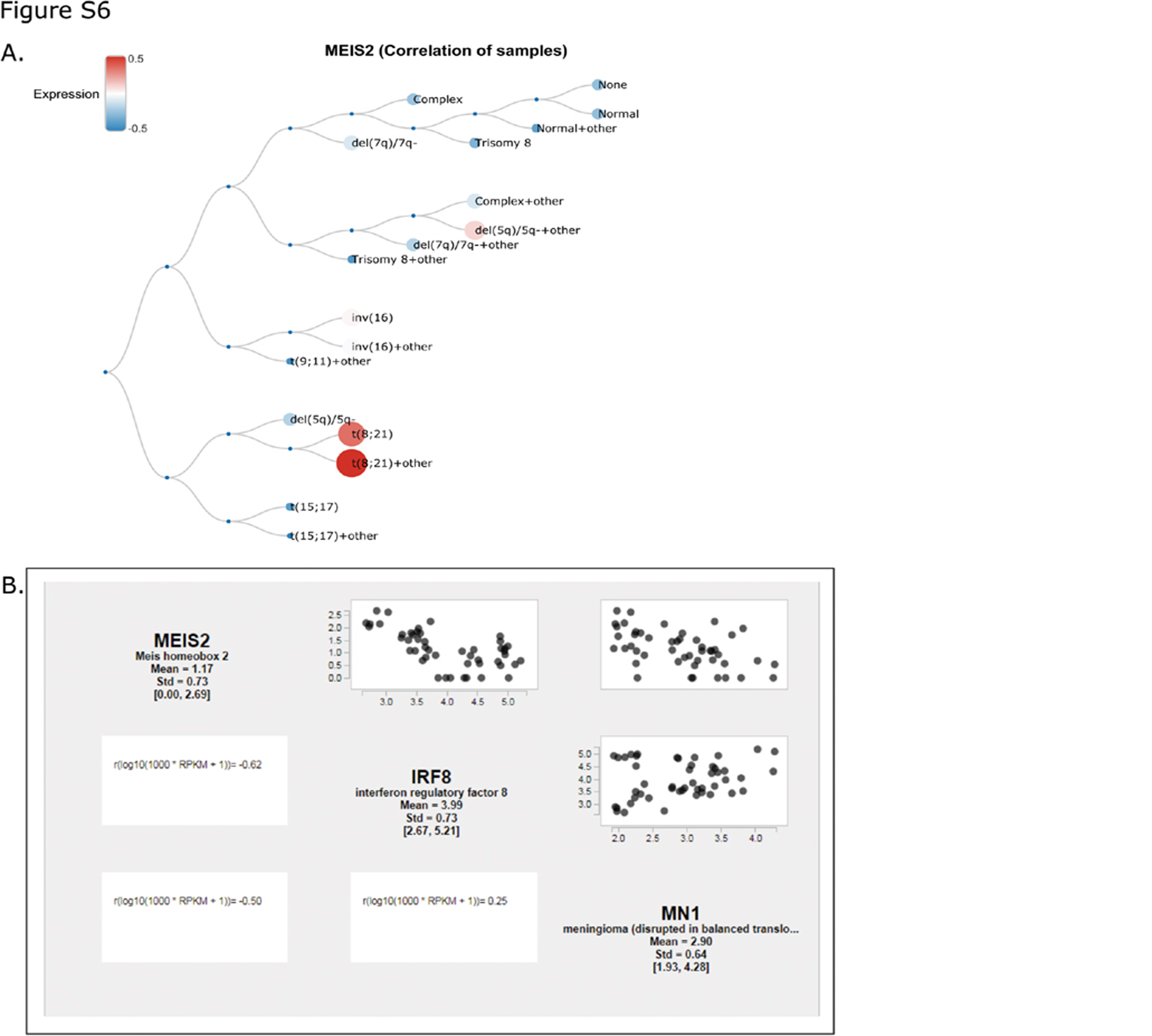

Supplement: Supplementary Figure S6 [file bcj201786x7.tif]
